# Patterns, Profiles, and Parsimony: Dissecting Transcriptional Signatures From Minimal Single-Cell RNA-Seq Output With SALSA

**DOI:** 10.3389/fgene.2020.511286

**Published:** 2020-10-09

**Authors:** Oswaldo A. Lozoya, Kathryn S. McClelland, Brian N. Papas, Jian-Liang Li, Humphrey H.-C. Yao

**Affiliations:** ^1^Genomic Integrity & Structural Biology Laboratory, National Institute of Environmental Health Sciences, National Institutes of Health, Research Triangle Park, NC, United States; ^2^Reproductive and Developmental Biology Laboratory, National Institute of Environmental Health Sciences, National Institutes of Health, Research Triangle Park, NC, United States; ^3^Integrative Bioinformatics Support Group, National Institute of Environmental Health Sciences, National Institutes of Health, Research Triangle Park, NC, United States

**Keywords:** scRNA-seq, NGS, RNA, single cells, heterogeneity, sparsity, reproducibility, hypothesis generation, transcriptomics analysis, biomarker discovery and validation

## Abstract

Single-cell RNA sequencing (scRNA-seq) technologies have precipitated the development of bioinformatic tools to reconstruct cell lineage specification and differentiation processes with single-cell precision. However, current start-up costs and recommended data volumes for statistical analysis remain prohibitively expensive, preventing scRNA-seq technologies from becoming mainstream. Here, we introduce single-cell amalgamation by latent semantic analysis (SALSA), a versatile workflow that combines measurement reliability metrics with latent variable extraction to infer robust expression profiles from ultra-sparse sc-RNAseq data. SALSA uses a matrix focusing approach that starts by identifying facultative genes with expression levels greater than experimental measurement precision and ends with cell clustering based on a minimal set of Profiler genes, each one a putative biomarker of cluster-specific expression profiles. To benchmark how SALSA performs in experimental settings, we used the publicly available 10X Genomics PBMC 3K dataset, a pre-curated silver standard from human frozen peripheral blood comprising 2,700 single-cell barcodes, and identified 7 major cell groups matching transcriptional profiles of peripheral blood cell types and driven agnostically by < 500 Profiler genes. Finally, we demonstrate successful implementation of SALSA in a replicative scRNA-seq scenario by using previously published DropSeq data from a multi-batch mouse retina experimental design, thereby identifying 10 transcriptionally distinct cell types from > 64,000 single cells across 7 independent biological replicates based on < 630 Profiler genes. With these results, SALSA demonstrates that robust pattern detection from scRNA-seq expression matrices only requires a fraction of the accrued data, suggesting that single-cell sequencing technologies can become affordable and widespread if meant as hypothesis-generation tools to extract large-scale differential expression effects.

## Introduction

Next-generation sequencing technologies are transforming how biologists characterize the molecular features of organogenesis and the composition of heterogeneous tissues; among them, RNA sequencing (RNA-seq) is one of the most widely adopted modalities (Mortazavi et al., 2008; Oshlack et al., 2010; Roy et al., 2011). RNA-seq on cell lines, sorted primary cells, and bulk tissues can be used to understand how transcriptional networks regulate cell fate determination and lineage specification during organogenesis, development, and disease (Cloonan et al., 2008; Gong et al., 2014; Oikawa et al., 2015; Li and Bushel, 2016; Li et al., 2017; Huynh et al., 2018). Yet, although bulk RNA-seq experiments have sufficed to determine gene expression signatures that underlie whole-organ physiology, they are inadequate to distinguish critical transitions in cell type-specific transcriptional dynamics, as they do without the inherent variation of gene expression across individual cells.

The traditional approach to interrogate transcriptional heterogeneity in tissues by RNA-seq relies on purifying subpopulations of collected cells (McClelland et al., 2015). However, this can be done only if relevant markers are known for each cell type in advance. It is also known that transcriptional output in single cells is exquisitely sensitive to how they are handled, meaning that the averaged transcriptome of a sorted cell subpopulation based on stable lineage markers may not match their gene expression dynamics *in vivo* (van den Brink et al., 2017). Single-cell transcriptomics circumvents many of these obstacles. A diverse catalog of single cell RNA-seq (scRNA-seq) platforms and workflows is available today, and still growing, that help reconstruct cell types and lineage specification processes in heterogeneous tissues at the level of individual cells (Picelli et al., 2013, 2014; Klein et al., 2015; Macosko et al., 2015; Cao et al., 2017, 2018; Rosenberg et al., 2018). Using bioinformatic tools, data from individual cells is deconstructed, sorted by gene expression similarities, and used to infer underlying cell types based on patterns of transcriptional signatures and functional ontology, directly from dissociated tissues, and without prior cell sorting or biomarker knowledge (Trapnell et al., 2014; Satija et al., 2015; Briggs et al., 2018; Farrell et al., 2018).

Still, with access to numerous customizable single-cell techniques comes new challenges for researchers on analysis of scRNA-seq data, chief among them data sparsity. In this work, we introduce a workflow, named single-cell amalgamation by latent semantic analysis (SALSA), that extract patterns of gene expression and single cell clusters from scRNA-seq datasets by leveraging their inherent sparsity. We benchmarked the cell type discriminative power of SALSA against the publicly available and widely regarded PBMC 3K standard, a single-run scRNA-seq reference dataset produced by 10X Genomics from human frozen peripheral blood (Zheng et al., 2017). After confirming that PBMC 3K is a scRNA-seq dataset with an ultra-sparse gene-cell expression matrix, we show how SALSA prioritizes gene data using statistical reliability metrics. Then, SALSA anchors clustering and differential expression analysis to a subset of genes with the most robust measurement features, which we call Profiler genes, and detects expression patterns that match the transcriptional signatures and relative abundance of cell types found in peripheral blood. Most importantly, we show that the Profiler gene fraction is sufficiently informative to identify the expected composition of blood cell types in PBMC 3K. By extension, we conclude that biological insight from similar scRNA-seq datasets may be at hand once sparsity is accounted for, and demonstrate it further by applying SALSA to integrate scRNA-seq data across multiple specimens in an unsupervised manner using Macosko’s DropSeq mouse retina dataset as test case (Macosko et al., 2015).

As we interpret it, the task at hand from the perspective of an experimenter performing scRNA-seq assays has less to do with establishing an expression atlas, and more to do with defining the most robust markers to recognize newly identified cell subpopulations in heterogeneous tissues. If that goal is attainable using the littlest amount of information possible, then scRNA-seq can be repurposed to yield manageable numbers of cell type-specific marker candidates quicker and with leaner sequencing expenses than in current practice; doing so affords small research groups with the ability to both embark in single-cell sequencing technologies and perform orthogonal confirmatory assays (e.g., PCR panels, ISH) that validate their findings. In this context, bridging the practical gaps between scRNA-seq bioinformatics, assay affordability, and experimental practice requires analytical workflows that prioritize information maximization rather than expression matrix completeness—SALSA being one possible embodiment of such core philosophy.

## System and Methods

### Publicly Available PBMC 3K Dataset From 10X Genomics

Count-level scRNA-seq data for peripheral mononuclear blood cells of a healthy human subject retrieved from a commercially available frozen stock (Zheng et al., 2017) is available for download from 10X Genomics^1^. Further details on scRNA-seq library assembly process, sequencing data acquisition, and single-cell barcode discrimination pipelines are available in the original publication by Zheng et al. (2017). For our analyses, we used a consensus curated version of the PBMC 3K dataset, available online courtesy of Rahul Satija’s research group at: https://s3-us-west-2.amazonaws.com/10x.files/samples/cell/pbmc3k/pbmc3k_filtered_gene_bc_matrices.tar.gz.

### Publicly Available Mouse Retina scRNA-Seq Dataset via DropSeq

Raw data was retrieved from NCBI Gene Expression Omnibus (GEO) under accession GSE63473 (Macosko et al., 2015) and processed into create an unfiltered gene × cell expression matrix using Seurat (Macosko et al., 2015; Satija et al., 2015).

## Algorithm

### A Probabilistic Mixture Model Finds Informative Subsets Within scRNA-Seq Expression Matrices

**FIGURE 1:**
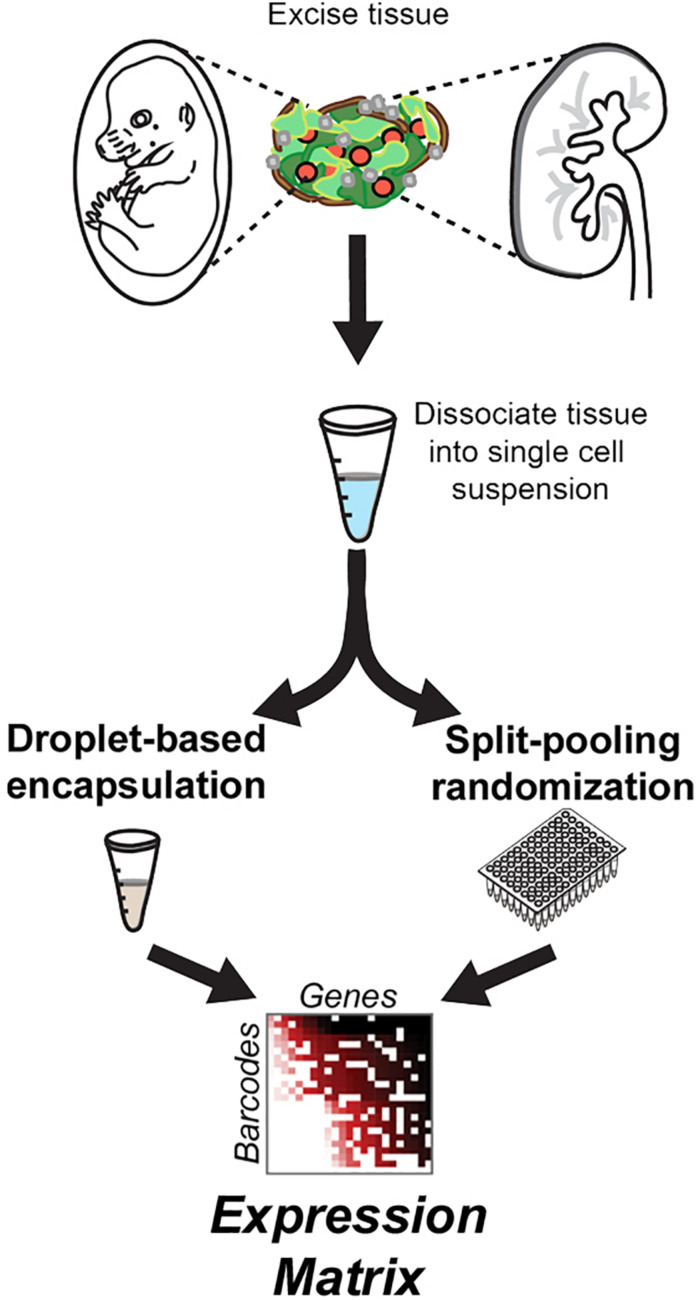
Basic steps of tissue-to-data process for massively paralleled single-cell RNA-seq technologies. To assemble an expression matrix for thousands of cells in a single run, biological specimens are dissociated into single cell suspensions, partitioned for barcoding and adapterization by droplet encapsulation (e.g., DropSeq) or split-pooling approaches (e.g., sci-RNA-seq), and sequenced with short-read high-throughput SBS instrumentation.

**FIGURE 2:**
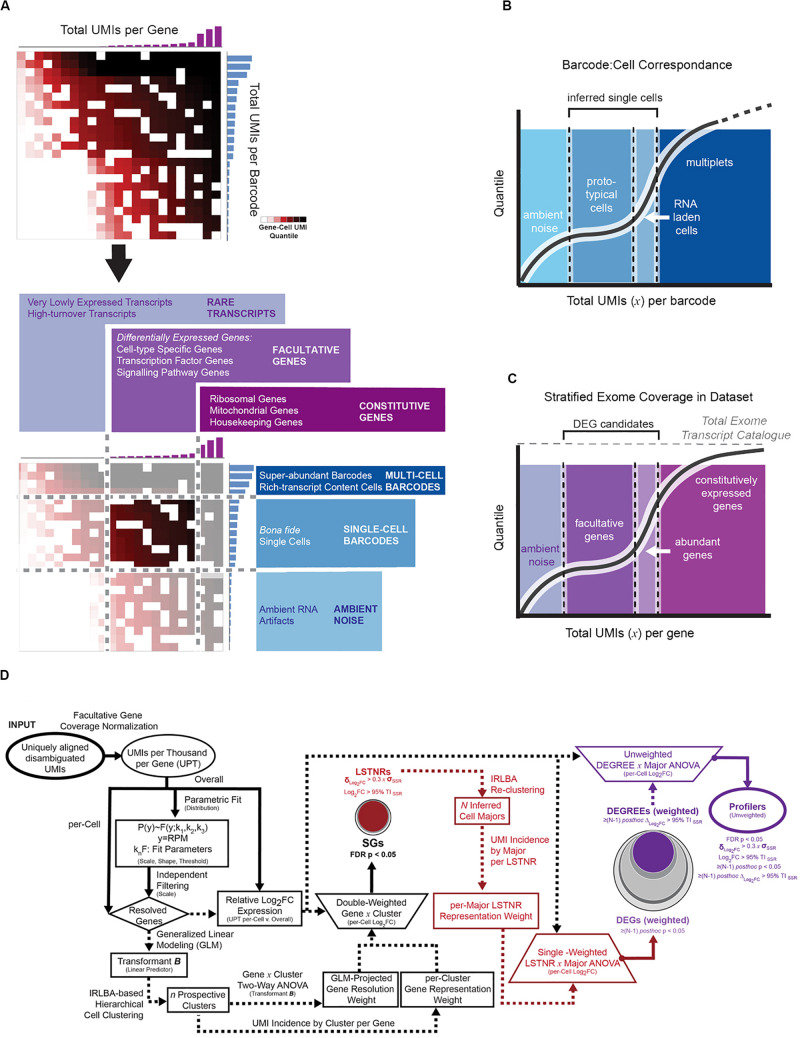
Depiction of expression matrix focusing by total per-gene and per-barcode coverage with the parametric P_C_-P_D_ mixture model. **(A)** Sorted count data from scRNA-seq experiments exhibits transitions in total UMI counts per barcode, reminiscent of distinct regimes of UMI density between background (ambient noise), single-cell, and multi-cell barcodes; total UMI counts per gene exhibit an analogous profile, with distinct regimes between rare, facultative, and constitutively expressed genes. Latent patterns of expression within gene-cell matrices are most discriminative at the intersection of facultative genes and single-cell barcodes regimes, referred to as the focused expression matrix. To infer coverage regimes per barcode **(B)** and per gene aligned **(C)** from the raw gene-cell expression matrix, total UMI count data are fit to a 2-component mixture probabilistic parametric model; regime thresholds are defined systematically from estimated scale and shape parameters. **(D)** Stratified differential expression analysis starting from a focused expression matrix in SALSA. The flow chart depicts transformations used in SALSA toward generalized linear modeling (GLM) of expression data, and statistical criteria to extract significant gene subsets with rising statistical stringency.

In most instances, massively paralleled scRNA-seq data is produced using droplet-based encapsulation or split-pooling methods, resulting in highly dimensional datasets known as expression matrices, consisting of tallied unique molecular identifiers (UMIs), which correspond to individual cDNA starting templates, per sequenced gene and per detected barcode (Figure 1). Then, the first step in scRNA-seq analysis is to infer which detected barcodes represent single-cell data. In all types of scRNA-seq pipelines, barcodes are deemed as single-cell flags based on context: one cell has less mRNA molecules than multiple cells, and therefore a single-cell barcode should be found in less cDNA templates than a multi-cell barcode. In turn, starting from a minimally degraded specimen, a barcode representing data from a single cell should encompass more UMIs than a barcode with data derived only from nucleic acid debris found in the cell suspension medium. As long as the cDNA yield in single cells is greater than the density of ambient debris in the cell suspension medium, distinguishing between artifactual, single-cell, and multi-cell barcodes should be able to rely on the disparate apportionment of total UMI counts among them (Figure 2A).

A useful barcode curation strategy should be widely applicable for scRNA-seq data from cells compartmentalized by different techniques. In the past, extreme event models have found broad applications in diverse research fields, including computational thread scheduling (Nair et al., 2010) and financial forecasting in econometrics (Cont, 2001), in which recognizing the advent of extreme events as they arise is key to decision-making. In such models, low-valued events are predominant, high-valued ones are rare, and their probabilistic spreads can be parametrically described as functions of the inflection point (scale parameters) and speed of transition (shape parameters) when moving between low and high values in the distribution of events (Supplementary Figures S1A,B). We deduced the behavior of total UMIs per barcode in sc-RNAseq datasets could match features of extreme value probabilistic models, and recognized at least two instances in which extreme value theory could be invoked: multi-cell barcodes are “rare events” relative to single-cell barcodes on the high-end of total UMI counts; and single-cell barcodes are “rare” relative to ambient artifacts at low UMI counts (Figure 2A). If so, we inferred, a mixture model of 2 or more extreme value distributions combined, each predominant in different scales of UMI tallies, could be used as an empirical parametric descriptor of total UMI counts per cell (or per gene) for the scRNA-seq dataset altogether. With this in mind, we defined a general two-component mixture distribution, the P_C_-P_D_ mixture model (Supplementary Figure S1A), that bridges two extreme scenarios to expect from different scRNA-seq techniques: (a) a finite number of barcodes is available, and all detected artifact and single-cell barcodes share a similar baseline level of UMI counts derived from nucleic acid debris throughout the biological specimen (“noise lifts barcodes,” akin to combinatorial based scRNA-seq techniques, Frechét distribution); and (b) there are substantially more artifact barcodes with low total UMI counts than single-cell barcodes with higher total UMI counts (“noise gets barcodes,” akin to droplet-based scRNA-seq techniques, Weibull distribution). Following quantile regression of total UMI counts per barcode to a parametric 2-component Weibull-Frechét mixture model and a heavy-tailed Frechét model, best-fit P_C_-P_D_ scale and shape parameters are combined algebraically to project lower and upper bounds for single-cell total UMI coverage, which estimates the boundaries between barcodes representing artifacts, cell singlets, and cell multiplets (Figures 2A,B and Supplementary Figure S1B). Using a similar logic, we use the same approach to segregate facultative genes from rare or constitutively expressed ones (Figures 2A,C). From here on in the analysis, and after having removed “extreme” tallies that disproportionately weigh on the information density inside the scRNA-seq expression matrix, we focus exclusively on data from “best-guess” single-cell barcodes and facultative genes to perform downstream unsupervised clustering and differential expression analysis. A detailed description of count-level data treatment using the P_C_-P_D_ mixture model is found in Supplementary Material.

### Differential Expression Analysis of scRNA-Seq Data Using Single-CELL AMALGAMATION by Latent Semantic Analysis (SALSA)

At its core, the SALSA methodology (Figure 2D) prioritizes information from *facultative* genes (those most likely to vary between individual barcodes) and projects it into multivariate space as an imputable eigenvalue problem. To do so, expression levels of individual genes (Ensembl annotation) in individual cells are calculated as the normalized rate of deduplicated and uniquely aligned UMIs-per-thousand total (UPT) per cell. Then, SALSA calculates “bulk” expression levels of each facultative gene (i.e., all single cells added together) to use as a “reference mean,” extract a best-fit parametric threshold distribution of expression intensities from the exponential family of distributions, and fits them against single-cell UPT rates to determine a linear predictor «***B***(**θ**)» of single-cell expression scores via generalized linear modeling (Nelder and Wedderburn, 1972). Once transformed into normally distributed linear predictors, expression scores can be interrogated further using multivariate analysis and latent pattern detection tools in common practice. SALSA defines prospective cell clusters based on «***B***(**θ**)» scores via an implicitly restarted Lanczos bidiagonalization algorithm (IRLBA) coupled with Euclidean hierarchical clustering (Ward’s method) (Baglama and Reichel, 2005), and then carries out differential expression analysis between the resulting clusters. Statistical tests of differential gene expression are performed using a two-way ANOVA model (gene × cluster blocks) of log2-transformed fold changes in single-cell UPT rates (Log_2_FC) relative to the gene reference mean, weighted for both resolution of mean gene coverage (such as in the LSTNR method; Lozoya et al., 2018) and for an often overlooked parameter: gene representation rates within clusters. Within-cluster gene representation rates are defined as the ratio of cells with aligned UMIs vs. total cells within a cluster for each gene. Gene-wise significance of Log2FC variation based on double-weighed ANOVA tests are adjusted by the Benjamini-Hochberg method for multiple comparisons (Benjamini and Hochberg, 1995).

We argue it is critical to consider gene representation rates when analyzing scRNAseq data because the meaning of “differentially expressed gene” in bulk vs. single-cell scales is fundamentally different. Most scRNA-seq data sets exhibit gene-cell matrices that are not only characterized by their sparsity (Mohammadi et al., 2018) but also by low gene × cell UMI counts. For example, knowing whether a target gene is expressed in similar frequency among cells from two separate cell subpopulations can be more informative than estimating whether transcript abundance among expressing cells between both groups is statistically significant. Without accounting for gene representation, such a scenario can go unnoticed in scRNA-seq analyses, particularly if the cells from both groups express similar numbers of overall transcripts per cell (equal *denominators*) and the target gene is transcribed in similar abundance among expressing cells regardless of group (equal *numerators*). Moreover, in cases where UMI coverage differs substantially between cell subpopulations, gene representation rates help balance statistical comparisons to distinguish whether inferred expression differences derive from true discrepancies in expression rates per cell (different *numerators*) or simply reflect overt normalization bias (different *denominators*).

**FIGURE 3:**
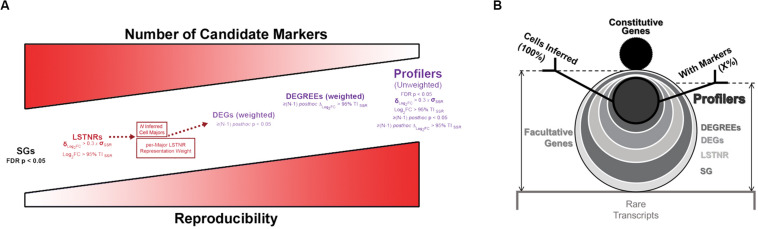
Facultative gene stratification with the SALSA workflow. **(A)** Progressive facultative gene strata with increasing levels of prospective experimental reproducibility. **(B)** Frosty plot of gene stratification across rising levels of statistical significance. Head (black circle, top), body (largest encasing circle, middle), and base (dim gray rectangle, bottom) depict the make-up of detected genes from a single-cell library based on their constitutive, facultative, or rarely expressed status; number of facultative genes admitted past significance criteria in each stratum are also shown as encasing circles with varying sizes and grayscale intensities. Stick arms flag the gene stratum chosen as the agnostic expression marker gene set for final inferential clustering of single-cell barcodes into cell majors. Retention rates of input and output single-cell barcodes following gene stratification are represented by the relative heights of stick arms going from 100% of inferred cells with facultative gene data (left arm) to a subset of inferred cells expressing agnostic markers.

Without gene prioritization criteria, it is difficult to anticipate which statistically significant differences in gene expression levels are most likely to elicit a functional outcome, can be replicated by independent scRNA-seq assays, or reproduced using orthogonal validation techniques. In SALSA, we address this challenge by sifting SGs through increasingly stringent filters of statistical significance, including “stress tests” against dynamic ranges of gene expression measurements, gene representation rates, and mutual exclusivity tests of expression between cell clusters (Figures 2D, 3A). For example, we define a signal-to-noise ratio threshold (SNR = 1) equal to the 95% tolerance interval (95% TI_*SSR*_) of log-fold expression residuals around means of prospective cell clusters. With it, SALSA can identify leveraged signal-to-noise ratio genes (LSTNRs) as those SGs with mean log-fold expression levels at SNR > 1 in at least one cell cluster. Going further, LSTNRs can then be stratified into DEGs (i.e., LSTNRs with pairwise significant differences between clusters), DEGREEs (DEGs with reproducible expectation estimates, with differences between cell majors greater than SNR = 1) and finally Profiler genes (DEGREEs that are still statistically significant even when the effect of gene representation rates per cluster is ignored).

As differentially expressed genes are sifted through increasingly stringent filters of statistical significance, “true” DEGs with higher probability of replication in independent experiments are retained, and “anecdotal” DEGs particular to a specimen or experimental batch lose statistical support and drop out along the SALSA workflow. At the same time, random effects of sequencing noise are muted. In the end, this gene stratification results in Profiler genes with large-scale effect sizes, either because they are only expressed in specific cell clusters, or because normalized expression levels between clusters with matching gene representation rates are quantitatively distinct. Profiler gene sets from SALSA are usually smaller than gene sets other pipelines report, which is advantageous for two substantial experimental purposes: being a small set of genes, validation of scRNA-seq data around Profilers is affordable; and being statistically significant independent of gene representation rates, Profilers are prospective biomarkers with a large probability of success in validation assays using bulk specimens. We convey gene stratification results hereafter using a short-hand graphical aid, the “frosty” plot, that illustrates the transition in data retention across filters of rising statistical stringency (Figure 3B).

## Results

### Validation With PBMC 3K, a Standard scRNA-Seq Reference Dataset

#### PBMC 3K Exhibits Near-Unary Architecture

To evaluate SALSA, we analyzed a publicly available “silver” standard dataset that is widely regarded for its single-cell coverage richness: the frozen Peripheral Blood Mononuclear Cells data set with 2,700 barcodes (or PBMC 3K) available through 10X Genomics. This dataset was originally produced by 10X Genomics from a single Illumina NextSeq 500 high-output flow cell run (Zheng et al., 2017). As is, the PBMC 3K set is available in a pre-filtered fashion, in that each of the represented 2,700 barcodes is presumed to represent a single cell. Overall, UMIs from single-cell barcodes aligned to 16,634 genes (hg19 reference).

**FIGURE 4:**
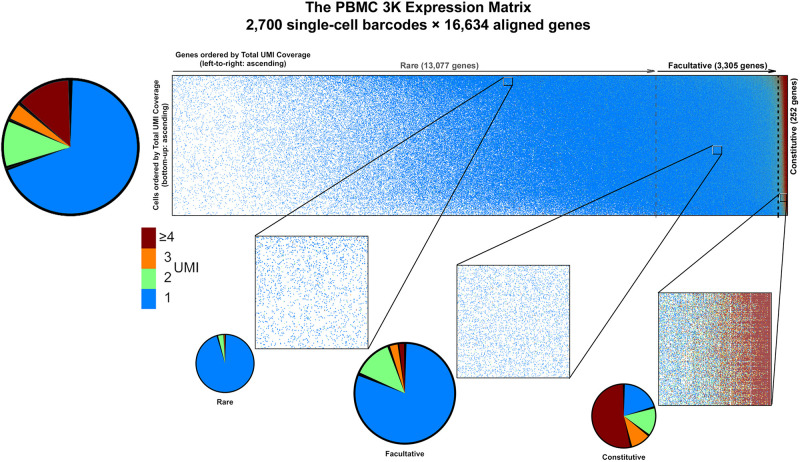
Graphical representation of data sparsity in the PBMC 3K expression matrix. Dots in the large rectangular frame (top) represent individual count values throughout the gene-cell expression matrix based on accrued sequencing data; missing data fields are blank. Vertical dotted gray lines demarcate the estimated boundaries between rare, facultative, and constitutively expressed genes. Make-up of count data values among data-positive fields (top left), with blow-up windows of ∼300 genes × 230 cells each (bottom) in the PBMC 3K expression matrix for rare, facultative, and constitutive gene regimes at high, middle, and low per-cell total UMI coverages, respectively.

PBMC 3K has a maximum allocation, or span, of 2,700 barcodes × 16,634 genes = 44.9M available spaces for non-zero UMI tallies in the gene-cell matrix. Notably, PBMC 3K contained a grand total of 6,390,631 barcode × UMI combinations which, once tallied, correspond to ∼2.3M barcode × gene data-positive UMI counts—accounting for only ∼5.1% of the available span (Table 1). Such data-positive fraction of PBMC 3K, composed of tallies of 1 or more UMIs per barcode × gene combination, was not strewn uniformly in the gene-cell matrix. For example, of the ∼2.3M data-positive fraction in the PBMC 3K gene-cell matrix, approximately 70, 12, 4, and 14% had counts of 1, 2, 3, and 4, respectively (Figure 4). Also, 1-valued barcode × gene UMI tallies contained alignments to 99.7% of all detected genes (16,588 genes), whereas only 23.6% of detected genes (3,929 genes) were represented in 4+-valued data-positive fields—with an astounding 84% of those 4+-valued tallies stemming from UMI alignments to only ∼1% of all detected genes (166 genes). Among those 166 “overrepresented” genes we found 8 protein-coding mtDNA genes, 75 ribosomal protein subunits, 8 HLA chains, and housekeeping genes like β-actin, GAPDH, and vimentin (Supplementary Table S1).

**TABLE 1:** Sparsity analysis of the PBMC 3K silver standard dataset by gene stratum.

Total UMI detected	6,390,631			
Gene-cell matrix span (16,634 × 2,700):	44,911,800			
**Data stratum (gene × cell block size | span)**	**# fields**	**% matrix**	**% stack**	**% block span**

**Total (16,634 × 2,700 | 44,911,800)**	**2,286,884**	**5.1%**	**100%**	**5.1%**
Constitutive genes (252 × 2,700 | 680,400)	536,804	1.2%	23.5%	78.9%
Facultative genes (3,305 × 2,700 | 8,923,500)	1,308,249	2.9%	57.2%	14.7%
*SGs (2,519* × *2,700 | 6,801,300)*	*1,046,003*	*2.3%*	*45.7%*	*15.4%*
*LSTNRs (2,519* × *2,700 | 6,801,300)*	*820,191*	*1.8%*	*35.9%*	*12.1%*
*DEGs (1,244* × *2,700 | 3,358,800)*	*558,892*	*1.2%*	*24.4%*	*16.6%*
*DEGREEs (464* × *2,700 | 1,252,800)*	*209,238*	*0.5%*	*9.1%*	*16.7%*
***Profilers (462* × *2,700 | 1,247,400)***	***209,089***	***0.5%***	***9.1%***	***16.8%***
Rare transcripts (13,077 × 2,700 | 35,307,900):	441,831	1.0%	19.3%	1.3%

*For reference, total accrued data volume metrics are shown (top row, bold) to compare against the Profiler gene stratum fraction used for cell type clustering (penultimate row, bold).*

In general, scRNA-seq data like PBMC 3K are compiled into gene-cell expression matrices which are sparse, dominated by low-count UMI tallies, and incompatible with traditional multivariate analytical methods or bulk RNA-seq analysis pipelines. In our view, such features of scRNA-seq expression matrices are best handled by dynamic sparsity-tackling algorithm such as IRLBA, which is designed to handle indexed data in stacked format (Baglama and Reichel, 2005). In the case of PBMC 3K analysis with SALSA, our approach meant retaining only the ∼2.3M data-positive barcode × gene UMI counts for analysis, a mere ∼5.1% of the data footprint required by a traditional zero-filled gene-cell expression matrix in other workflows.

#### Expression Matrix Focusing of PBMC 3K by Parametric Sweeping

**FIGURE 5:**
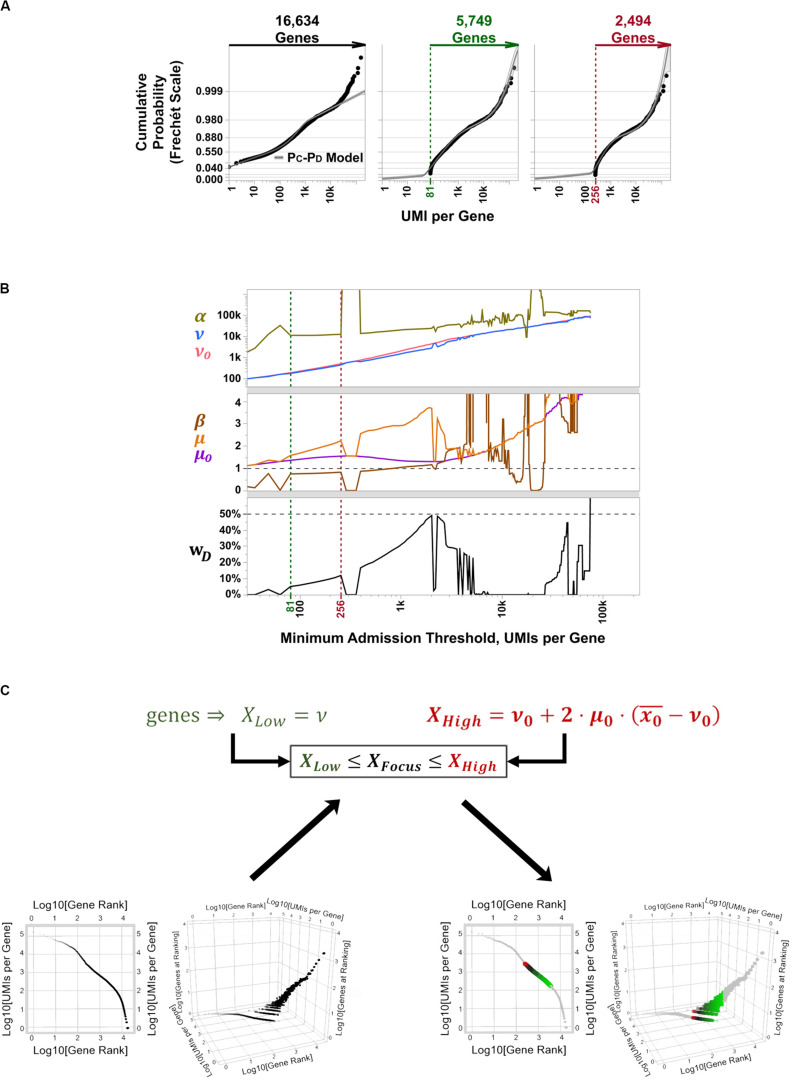
Parametric sweeping implementation for facultative gene extraction from the PBMC 3K silver standard dataset. **(A)** Example quantile plots for P_C_-P_D_ mixture model fitting at varying minimum coverage admission thresholds over all 16,634 aligned genes: no threshold (left, black label lettering), 5,749 aligned genes with > 81 total UMI counts (middle, green label lettering), and 2,494 with > 256 total UMI counts (right, red label lettering). **(B)** Parametric sweep with rising minimum coverage admission thresholds per gene of P_C_-P_D_ and heavy-tailed projection models. Green and red vertical dotted lines demarcate the span of best-fit parameters for the inferred facultative gene regime, flanked by numerical solver instabilities, that correspond to quantile plots in **(B)** with matching label colors. **(C)** Traditional gene knee plot displays of the PBMC 3K data set with an additional z-axis showing numbers of genes sharing ranking positions (log-scale), showing all detected genes (bottom left) and highlighting inferred facultative genes (bottom right) through matrix focusing. Inferred facultative genes are shown in a low-to-high total UMI coverage color gradient (green-to-black-to-red; bottom right).

To determine the best candidate subset of highly variable genes to use for cell type discrimination and differential expression analysis in PBMC 3K, we tallied and recorded aggregate UMI counts per aligned gene, ranked them between those with low overall detection rates (i.e., rare transcripts) and extraordinarily high counts at “outlier levels” across the board (i.e., constitutive genes), and fit their probabilistic spread to our P_C_-P_D_ mixture model to implement matrix focusing based on per-gene coverages (Supplementary Figure S1A). In this approach, the subset of facultative genes is then chosen by parametric sweeping as follows: an empirical cut-off for the minimum gene coverage considered informative is imposed, a best-fit distribution regression is performed on the coverage rates of admitted genes, and best-fit P_C_-P_D_ parameter estimates are recorded; then, the coverage cut-off is raised, and a new set of best-fit parameters are estimated and recorded (Figure 5A). After sweeping through all gene coverage values, estimated P_C_-P_D_ parameters are plotted across iterations. The plots of evolving P_C_-P_D_ parameter values vs. their respective coverage cut-offs are explored for “spikes,” which highlight steep transitions in coverage values from rare, facultative, and constitutive genes (Figure 5B). Such spikes are expectable since the SALSA parametric sweep uses a continuous-valued best-fit regression to fit a discrete-valued empirical distribution—i.e., the spikes stem from numerical solver instabilities that occur when the admission cut-off lands in between genes whose coverage shifts suddenly. Last, best-fit P_C_-P_D_ parameters flanked by “spike” solutions are used to estimate the range of “inlier” per-gene coverages that correspond to facultative genes (Figure 5C). In PBMC 3K, each reported barcode has been scored as a single cell in advance; thus, no barcode filtering was needed for our analyses. Because of its parametric nature, we argue our filtering approach to recognize facultative genes can be implemented in a systematic way—without compromising on data individuality from independent scRNA-seq libraries.

Based on the P_C_-P_D_ parametric sweep of the PBMC 3K data set, we partitioned the 16,634 detected genes into three categories: 13,077 rarely aligned genes (1–168 total UMIs each); 3,305 facultative genes (169–2,799 total UMIs each); and 252 constitutive genes (2,814–161,685 total UMIs each) (Table 1 and Figure 4). The structure of the PBMC 3K data set was notable in that the data-positive fields were unevenly apportioned among the three gene coverage regimes. For example, rare genes portion of the matrix had a 95.8% rate of 1-valued count fields. SALSA labeled these transcripts as *rare* because they were detected few and far between, peppered throughout the matrix at frequencies reminiscent of indiscriminate sequencing artifacts, and presumably without apportionment bias among single cells. In contrast, the constitutive genes portion of the matrix was dominated by multi-count data-positive fields (20.6 vs. 53.7% rates of 1-valued vs. ≥ 4-valued count fields, respectively). This suggests that many of the constitutive genes were often, or always, detected multiple times in most, if not all, single cells. Designation of these genes as *constitutive* is also supported by the fact that each of the 166 genes designated earlier in the workflow as “overrepresented,” based on their predominantly multi-count data make-up, were parametrically assigned to this stratum.

Everything considered, the 3,305 × 2,700 facultative gene portion of the matrix accrued more data than the constitutive and rare gene portions combined. Facultative genes also showed an intermediate diversity in the make-up of count values with 81.3% of the data coming from 1-valued count fields, 13.5% for 2-valued count fields, and all other fields with 3 or higher UMI counts (Figure 4). In principle, these data features would suggest many genes in this subset were detected somewhat frequently among single cells. Genes in this subset have a gradient of count values—some cells express them, some do not, and some express the transcripts at rates that wax and wane.

We propose that parametric focusing of gene-cell matrices for the PBMC 3K data set, and arguably, for any scRNA-seq data sets, is a systematic curation strategy that favors retention of diverse blocks of single-cell expression data for subsequent analysis. This strategy for data curation strikes a balance between data volume, computational performance, and statistical variation. Of note, we did not perform parametric focusing at the barcode level on the PBMC 3K dataset because the source files we used only reported single-cell barcodes; even then, parametric focusing on genes alone identified gene subsets to withdraw from further analysis and greatly reduced the computational data load. In the PBMC 3K data set, SALSA reduced the data to be analyzed to a ∼1.3M UMI count stack vs. the original ∼45M zero-filled count matrix (Table 1). In practical terms, our parametric focusing approach to pre-processing raw scRNA-seq datasets efficiently distills the informative fraction of expression data from the prominently empty-valued matrix for further analyses.

#### SALSA Identifies Cell Types in PBMC 3K Using Data From Stratified Facultative Genes Alone

**FIGURE 6:**
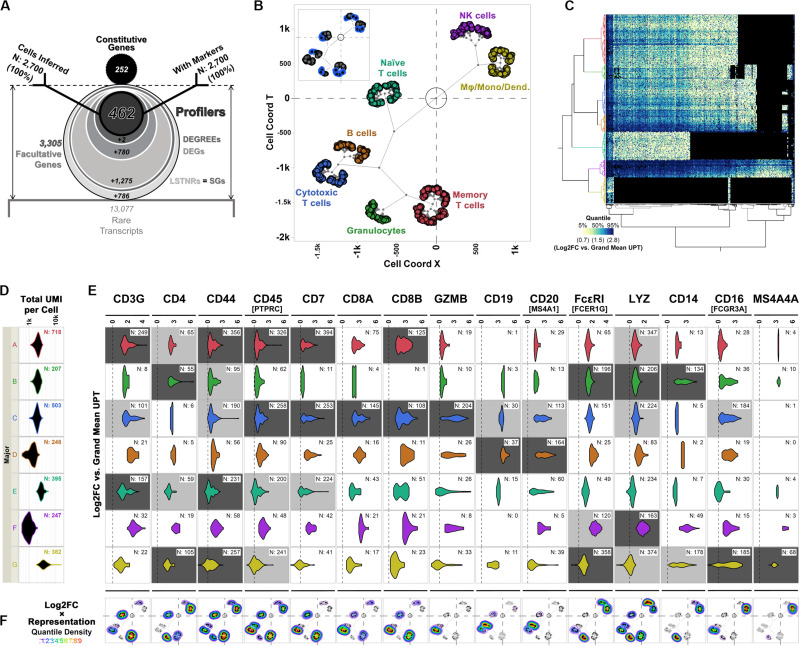
Differential expression analysis and cell type inferences in the PBMC 3K dataset using SALSA. **(A)** Frosty plot of gene stratification across rising levels of statistical significance in PBMC 3K. **(B)** Putative cell types matched to cell majors and their inferred transcriptional proximities displayed in latent 2D space by unsupervised clustering of mean linear predictor estimates «***B***(**θ**)» for expression rates of agnostic markers. **(C)** Heatmap overlay onto two-way clustering dendrograms from **(B)** showing increasing quantile scores of Log_2_FC values relative to library-wise UPT grand mean (tan-to-cyan-to-blue); missing data fields are shown in black. **(D)** Violin plots for total UMI coverage per barcode (*x*-axis) within cell majors; inset legends report total number of barcodes per cell major. **(E)** Violin plots of Log_2_FC values relative to library-wise UPT grand means (*x*-axis) for 15 landmark expression genes of blood cell types across cell majors in PBMC 3K; inset legends report total number of barcodes with UMIs for each landmark gene. Relative “yes/no” representation rates of landmark genes, i.e., the ratio of expressing vs. total cells within cell majors, are illustrated by coloring of violin plot backgrounds: in light gray, majors with high expression levels for a given landmark gene; in dark gray, majors with high expression levels and representation rates combined. **(F)** Topographs showing the patterns of expressed landmark gene enrichment across the latent 2D space map from **(B)**, overlayed with a non-parametric quantile heatmap highlighting “weighed gene expression” scores, i.e., the composite score of single-cell Log_2_FC values and within-major representation rates per gene; individual expressing cells are shown as black dots in 2D clustering maps.

After extracting facultative gene data from PBMC 3K, we used the SALSA workflow to infer distinct transcriptional groupings among detected cells and perform differential expression analyses. Briefly, based on facultative gene data alone, we identified 2,519 LSTNR genes (Table 1 and Figure 6A) among 7 prospective clusters without barcode dropouts (Table 1). These clusters were refined into “cell majors” by re-clustering cells based exclusively on LSTNR gene expression data (Figure 2D). Then, we recorded how often each LSTNR gene was detected in cells within a major, combined those representation rates with mean expression differences between clusters, and stratified LSTNR gene subsets as a function of their reproducibility potential in benchtop assays. A detailed description of our analysis progression is available in the Supplementary Discussion.

The frosty plot for the PBMC 3K data using SALSA-based gene stratification is shown in Figure 6A. By sequentially “stressing” statistical comparisons among cell majors from a starting list of 2,519 LSTNR genes, we sifted the pool down to: (a) 1,244 DEGs, a subset of LSTNRs whose net Log_2_FC pairwise differences between cell majors are statistically significant and mutually exclusive regardless of their location within the dynamic range of sequencing detection; (b) 464 DEGREEs, a subset of DEGs with statistically significant pairwise differences greater than the SNR = 1 noise benchmark between cell majors; and (c) 462 Profilers, a subset of DEGREEs with expression levels between cell majors that remain statistically significant even if gene representation rates between separate cell majors are ignored in the analysis. Notably, even though the number of retained gene × cell count data fields dropped as the number of genes decreased between strata (Table 1) our stratification approach led to a substantial improvement on information density. Ultimately the 462 × 2,700 Profiler block represents ∼0.5% of the gene-cell matrix allowance, however, this span is over 3-times more populated as a subset than the gene-cell expression matrix overall (outlined in Table 1). These results suggest that facultative gene stratification retains underlying transcriptional profiles of single cells, thereby pointing to SALSA successfully extracting a parsimonious subset of testable, agnostically defined candidate biomarkers.

To inspect if profiler-based unsupervised clustering reflected distinct signatures among peripheral blood subpopulations, we focused on expression data from a reference subset of 15 “landmark” genes encoding 14 widely recognized protein markers (Figures 6C,E,F and Table 2). We also devised “topographs” consisting of neighbor-joining trees that simultaneously highlight differences in the intensity and predominance of expressed genes among cell majors (Figure 6F and Supplementary Figure S2). Altogether, single-cell clustering based on Profiler genes in the 3K PBMC dataset revealed 7 distinctive single-cell clusters (Figures 6B,C) split into two broad transcriptome categories: the first one containing both the A-D ensemble and the intermediate E stem (2,071 cells), and the second one with the F-G stem (629 cells).

**TABLE 2:** Landmark genes, their gene stratum classification by SALSA, and their expression levels among cell types in the PBMC 3K silver standard dataset.

Landmark gene name [Entrez symbols]	Gene stratum	Naïve T-cell	Memory T-cell	Cytotoxic T-cell	B-cell	NK cell	Granulocytes	Monocyte and M-derivatives
CD3 [CD3D/E/G]	DEG	++	++	+				
CD4	Facultative	+	+				++	++
CD44	Profiler	+	++	+			+	++
CD45_*R*__*A/B/C/O*_ [PTPRC]	Profiler	+	++	++				+
CD7	Profiler	+	++	++				
CD8 [CD8A/B]	LSTNR		+	++				
GZMB	Facultative			++				
CD19	Rare				++			
CD20 [MS4A1]	Facultative				++			
FcεRI [FCER1A/G, MS4A2]	Constitutive					+	++	++
LYZ	Constitutive		+	+		++	++	+
CD14	LSTNR						++	++
CD16 [FCGR3A]	LSTNR			++				++
MS4A4A	Rare							++

The first transcriptome category hosted 1,864 cells with transcriptional signatures characteristic of lymphoid-derived T cells (majors A, C, and E) and B cells (major D); this contribution is consistent with the reported 4:1 ratio, for cells of lymphoid vs. myeloid origin in the source PBMC stock (Zheng et al., 2017). Cells in major B showed expression signatures corresponding with granulocyte functions cells (Hambleton et al., 1996; Shi et al., 2004; Wakabayashi et al., 2006; Bednar et al., 2014; Supplementary Figures S2, S3). When violin plots failed to highlight differences between majors A, C, and E in the PBMC 3K data set (Figure 6E), topographs performed better by simultaneously revealing log-fold expression, representation rates, and the location of expressing individual cells in clustering maps for a gene of interest (Figure 6F). By using topographs for landmark genes (Table 2) we recognized majors E, A and C as naïve, memory and cytotoxic T cells, respectively, this is in agreement with varying degrees of enrichment for additional T cell maturation markers (Supplementary Figure S2; Khattri et al., 2003; Yagi et al., 2004; Ahlers and Belyakov, 2010; Churlaud et al., 2015; Hu et al., 2018).

The second transcriptome category constituted majors F and G. Though similar to granulocytes (cell major B), cells in major G clustered apart and were also distinct in critical ways, primarily by their high expression levels of monocytic and macrophage-enriched genes CD16(FCGR3A) and MS4A4A, respectively (Figures 6B,C,E; Mandl et al., 2014; Sanyal et al., 2017; Hu et al., 2018). We concluded that cell major G represents a combined pool of monocyte-derived subtypes including monocytes, macrophages, and mono-derived dendritic cells. Finally, we surmised clustering proximity between majors F and G may have resulted from converging physiologies. In turn, we found cells in major F were best matched to lymphoid-derived natural killer (NK) cells based on some defining features: (1) a strong ontological relationship with monocytic cell types, (2) their relative frequency in the data set (∼9% of single cells), and (3) presentation of innate immunity signatures (Hanna et al., 2004; Gustafsson et al., 2008; Pokkali et al., 2009; Poli et al., 2009; Romee et al., 2013; Zheng et al., 2017).

Finally, the segregation of T cell subtypes, B cells, and antigen-presenting granulocytes under the same transcriptome category when using the SALSA workflow was consistent with an underlying and powerful biological feature: those four cell types constitute the adaptive immune system. Conversely, the second transcriptome category depicted the main players in the innate immune response: NK cells and monocyte-derived cells. We found that unsupervised clustering of single cells inferred by SALSA recapitulated the expression patterns of traditional marker genes and proportions of cell types expected from PBMC specimens without data preconditioning. Thus, by performing single-cell profiling on the “silver” standard PBMC 3K dataset, we demonstrate the core strengths of the SALSA workflow. With SALSA, a minimal fraction of well-resolved expression data from agnostic Profiler genes successfully sorted like cells, recapitulated experimentally demonstrated transcriptional signatures, and retained latent linkages that evoke converging physiologies among interconnected cell types.

### Salsa as an Integrative Workflow for Replicative scRNA-Seq Analysis

#### Macosko’s DropSeq Mouse Retina Dataset, a Reference Multi-Batch scRNA-Seq Experimental Design

From a reproducibility perspective, identifying candidate biomarkers from scRNA-seq experiments is best if data from multiple and independently sequenced specimens (i.e., biological replicates) from an experimental group can be integrated. Candidate biomarkers inferred from scRNA-seq that are detected in all biological replicates are also more likely to succeed in orthogonal validation assays. SALSA provides the means to refine the process of identifying candidate biomarkers from replicative assays even further: it can take independently sequenced scRNA-seq libraries, determine subsets of replicated genes ranking at different levels of prospective reproducibility for each—from facultative to profiler genes—and prioritize which commonly detected genes to include for an all-at-once scRNA-seq analyses.

To benchmark how such an integrative approach would perform in a replicative experimental setting, we analyzed publicly available scRNA-seq data from a mouse retina profiling study (Macosko et al., 2015). This dataset offers key advantages to test integrative performance of scRNA-seq workflows: it contains data from 7 individual DropSeq libraries, each assembled from an independent biological specimen, prepared across 4 experimental rounds (day 1: specimen 1; day 2: specimens 2 and 3; day 3: specimens 4, 5 and 6; day 4: specimen 7), and sequenced in separate NextSeq 500 high-output flow cells. Macosko’s retina dataset compiles > 108M total UMIs aligned to > 21,500 annotated genes (mm10 reference genome).

**FIGURE 7:**
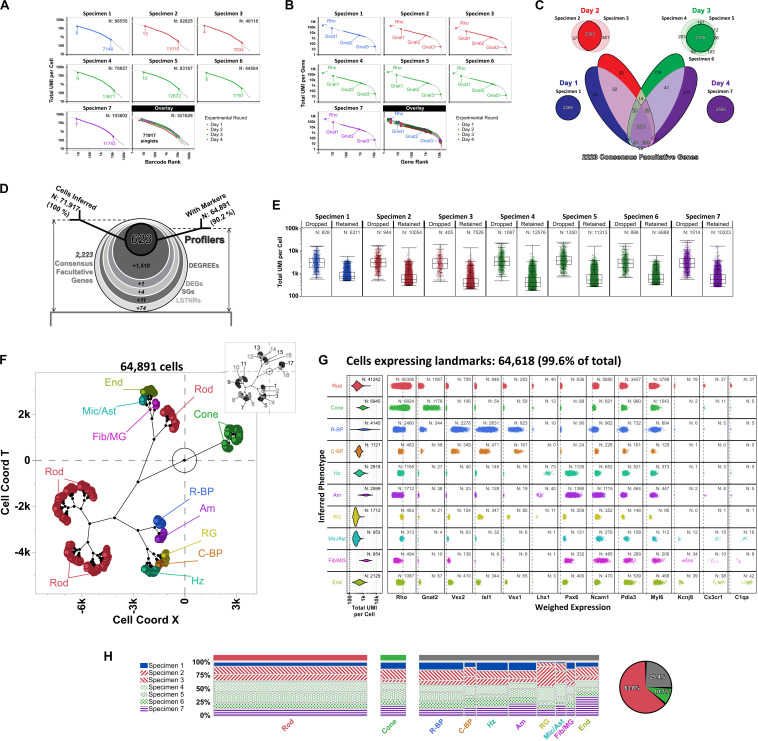
Differential expression analysis and cell type inferences in Macosko’s mouse retina DropSeq dataset using SALSA. Knee plots for **(A)** detected barcodes and **(B)** aligned genes from Macosko et al. (2015) dataset, highlighting inferred singlets and facultative genes by separate parametric sweepings within each specimen using the P_C_-P_D_ mixture model. Rankings corresponding to the highest- and lowest-count inferred singlets in barcode knee plots, as well as positions of *Gnat1*, *Gnat2*, and *Gnat3* in gene knee plots, are shown for each specimen separately. Plot colors depict specimens collected and processed in each of 4 separate experimental rounds; total barcodes and genes detected per specimen are shown within each knee plot (top right). **(C)** Stepwise selection of consensus facultative gene set used to implement cross-specimen integrative analysis of Macosko’s retina dataset by SALSA. **(D)** Frosty plot of gene stratification across rising levels of statistical significance of the integrated Macosko’s retina dataset. **(E)** Distribution of total UMI per cell rates in dropped vs. retained single cell barcodes per specimen after integrative gene stratification analysis using SALSA. **(F)** A set of 10 inferred retinal cell phenotypes across 64,891 retained single cell barcodes in latent 2D space (main plot; Rod, rod photoreceptors; Cone, cone photoreceptors; R-BP, rod bipolar cells; C-BP, cone bipolar cells; Hz, horizontal cells; Am, amacrine cells; RG, retinal ganglion cells; Mic/Ast, microglia and astrocytes; Fib/MG, fibroblasts and Müller glia; End, endothelial cells) in relation to 18 agnostically determined cell majors (inset, grayscale). **(G)** Violin plots for 64,618 single cell barcodes expressing retinal cell markers. Left-most column: total UMI coverage per barcode; inset legends report total number of barcodes per phenotype. Rest: weighed expression levels across 13 landmark genes relative to library-wise grand means (*x*-axis, dashed line); inset legends report total number of barcodes with UMIs per phenotype for each landmark gene. **(H)** Contingency plots for contribution per specimen to each inferred cell phenotype; far right: overall fractions among 64,618 landmark-expressing cells of rod, cone, and all other retinal cells combined.

In dissecting Macosko’s retina dataset, we identified a subset of 14,472 protein-coding non-ribosomal genes that harbored UMIs from all independently sequenced libraries (range of total protein-coding non-ribosomal genes aligned per specimen: 17,959–19,154; median: 18,356). Data from the replicated 14,472-gene subset were spread across 521,628 barcodes overall (range of total barcodes per specimen: 40,118–103,602; median: 83,167). For our handling of the data, we did not assume that UMI tallies were distributed equivalently between independent libraries in our analyses; instead, we determined a list of inferred singlets to include in subsequent analyses by performing P_C_-P_D_ parametric sweeps on total UMIs per barcode for each specimen separately. Using this stratified approach, we inferred 71,917 singlets overall (Figure 7A), with a total ∼69.5M UMIs contained within the replicated 14,472-gene subset and arranged into ∼41.7M non-zero gene × barcode UMI tallies (1-valued: 72.2%; 2-valued: 16.4%; 3-valued: 5.3%, 4 + -valued: 6.1%). Based on these metrics, our analytical space for Macosko’s retina dataset started as a 14,472 × 71,917 gene-cell expression matrix with an occupancy rate of 41.7M ÷ [14,472 × 71,917] ∼4.0%. Within this analytical gene-cell expression matrix, 1-valued UMI tallies (∼30.1M data-positive fields) contained alignments to all genes in the replicated 14,472-gene subset; in contrast, 50% of all 4 + -valued UMI tallies (∼2.5M data-positive fields) stemmed from alignments to only 150 “overrepresented” genes (∼1% of replicated genes), with the rest of 4 + -valued UMI spread among 10,407 other genes (71.9% of replicated genes). As expected, ontological analysis by Enrichr (Kuleshov et al., 2016) using the 150 “overrepresented” genes correlated with enrichment of phototransduction-associated pathways, rhodopsin-mediated biological processes, and the expression atlas of retinal pigment epithelia in mice (Supplementary Table S2).

As reported originally (Macosko et al., 2015) we found that UMIs for *Rho* transcripts were ubiquitous among all inferred singlets, which matched with its ranking as a constitutive gene in all independent specimens. This observation is consistent with suspected solubilization of transcripts from rod photoreceptors, the most abundant cells in retina (∼65%), when preparing retinal cell suspensions. As a result, data from genes highly expressed in rod cells such as *Rho* are expected to “bleed-through” across the expression matrix; along the same lines, we also found the rod-specific α1-transducin gene *Gnat1* (Lin et al., 2013) displayed constitutive abundance (Figure 7B). In comparison, the α2-transducin gene *Gnat2*, a known cell-specific marker of cone photoreceptor cells (Lin et al., 2013; Ronning et al., 2018), ranked as a facultative gene across specimens, which can also be explained by the same logic used for *Rho* from rod cells but leading to significantly lower UMI totals due to the few numbers of cone photoreceptors overall in retina (∼4% of cells) (Jeon et al., 1998). As counterexample, the closely associated *Gnat3* gene, encoding the α3-transducin subunit, always ranked as a rarely aligned gene (Figure 7B), which is consistent with its known tissue-specific expression in taste receptors but not in the eye (McLaughlin et al., 1992). Altogether, these assessments support our matrix focusing strategy as a systematic means to prioritize variable and informative genes in both single- and multi-replicate scRNA-seq analyses, and discard detected transcripts from rarely aligned genes that may represent experimental or bioinformatics-derived artifacts.

Moving into cross-replicate integration, we honed our SALSA analysis toward a “consensus” subset of facultative genes (Figure 7C). The consensus facultative gene subset consisted of 2,223 replicated genes that: (a) ranked as “batch-consistent” facultative genes for all biological replicates from the same experimental round (e.g., gene is facultative in all specimens from day 3); and (b) repeated as “batch-consistent” facultative genes in most experimental rounds (i.e., in at least 3 out of the 4 days that DropSeq libraries were prepared). One advantage to this strategy is that it identifies facultative genes simply by carrying out P_C_-P_D_ parametric sweeps on total UMIs per gene for each independent specimen and requiring no further analysis. Another advantage is that it devotes computational resources to genes that score as facultative in a reproducible manner. Also, by representing an intersection of data from separate specimens, the 2,223-gene consensus facultative set is smaller than any of the specimen-specific ones (range of facultative genes per specimen: 40,118–103,602; median: 83,167) suggesting that experiments with more biological replicates make for leaner scRNA-seq analyses across increasingly reproducible genes. Finally, this stratified approach lowers the probability of bioinformatic inferences reflecting a bias toward gene expression data from botched biological replicates, either because relative contributions of facultative genes to the consensus set become glaringly obvious when a particular replicate is imbalanced compared to all others, or because genes with artificially (or artifactually) distorted representation rates in a particular replicate do not score frequently enough as facultative among the rest—i.e., they are anecdotal facultative genes, not reproducible ones.

As a group, the 2,223 consensus facultative genes were represented in all 71,917 inferred singlets (range of consensus facultative genes per inferred singlet: 9 – 2,096; median: 227), totaling ∼39.5M UMIs arranged into ∼23.6M non-zero gene × singlet UMI tallies (1-valued: 67.6%; 2-valued: 19.0%; 3-valued: 6.7%, 4 + -valued: 6.7%) for an occupancy rate of 23.6M ÷ [2,223 × 71,917] ∼14.8% within the consensus facultative block of the integrated expression matrix. Following gene stratification by SALSA (Figure 7D), we reduced the integrated expression dataset to 623 Profiler genes, expressed by 64,891 high-confidence inferred cells (i.e., 90.2% singlet retention rate). Conversely, this result also meant 7,026 initially inferred singlets dropped out from our analysis; upon further inspection, we found that inferred singlet dropouts within all specimens consistently accrued more UMIs (median UMIs per singlet within specimens: 2,767–3,638) than their retained counterparts (median UMIs per singlets within specimens: 372–757) (Figure 7E). Given their large differences in total UMIs per barcode compared to retained singlets, our analysis suggests that singlets dropped out after gene stratification with SALSA likely represent multi-cell barcodes that exhibit similar UMI counts for profiler genes than single-cell barcodes, but go on to fail signal-to-noise filtering because their normalized expression rates are overly “diluted” by their high UMI counts. This observation would also suggest single cell RNA-seq datasets contain apparent single-cell barcodes that are unrecognizable from high-confidence single-cell ones by total-UMI-per-cell diagnostics alone unless (and until) they are statistically sieved through signal-to-noise filters based on normalized gene expression rates. This is an important, but otherwise inconspicuous, distinction between high-confidence and apparent single-cell barcodes that SALSA and few (or no other) scRNA-seq processing methods currently available can discern, through agnostic and systematic gene stratification, by unsupervised scRNA-seq expression analysis.

Based on data from the 623 integrated profiler genes expressed among the 64,891 total cells we recognized in Macosko’s retina dataset [specimen 1: 6,311 cells (9.7% of total), specimen 2: 10,054 (15.5%), specimen 3: 7,526 (11.6%), specimen 4: 12,576 (19.4%), specimen 5: 11,313 (17.4%), specimen 6: 6,888 (10.6%), specimen 7: 10,223 (15.8%)] SALSA identified 18 agnostic cell clusters that matched transcriptional profiles of 10 distinctive and previously reported cell subpopulations in the mouse retina (Figure 7F; Macosko et al., 2015), as shown by the expression patterns of cell type-enriched landmark genes among 64,618 (99.6%) of the total cells integrated by SALSA (Figure 7G; Furukawa et al., 1997; Jeon et al., 1998; Koike et al., 2007; Puthussery et al., 2010; Sarin et al., 2018). Overall, we found that rod and cone photoreceptor cells accounted for the two most abundant subpopulations among cells expressing landmarks [Rod: 41,242 cells (63.8% of cells expressing landmark genes); Cone: 6,945 (10.7%)]; in term of relative abundance, photoreceptor cells were followed by rod bipolar [R-BP: 4,145 (6.4%)], horizontal [Hz: 2,918 (4.5%)], amacrine [Am: 2,599 (4.0%)], endothelial [End: 2,129 (3.3%)], retinal ganglion [RG: 1,712 (2.6%)], and cone bipolar cells [C-BP: 1,121 (1.7%)], and finally by two mixed-phenotype fractions: one expressing landmark genes for microglia and astrocytes [Mic/Ast: 953 (1.5%)]; and another expressing fibroblast and Müller glia signatures [Fib/MG: 854 (1.3%)].

In general, contributions to each cell type from independent specimens were commensurate to each specimen’s relative representation within the overall 64,618 integrated cells tally (Figure 7H). Still, we observed some discrepancies that depended on the cell type in terms of their relative contributions to the total cell tally; those discrepancies in cell type-specific contributions traced back to specific experimental rounds, and included Rod and C-BP in the sample from day 1 (underrepresented relative to other inferred cell types from that specimen preparation batch), RG and Mic/Ast (mostly absent from day 1 data), as well as Cone, Am, and End cells (disproportionally sourced from the single day 4 specimen) (Figure 7H).

Altogether, the final 623 × 64,891 profiler block from the integrated expression matrix comprised ∼7.2M UMIs arranged into ∼5.2M non-zero gene × cell UMI tallies (1-valued: 75.3%; 2-valued: 17.8%; 3-valued: 4.6%, 4 + -valued: 2.3%) for an occupancy rate of 5.2M ÷ [623 × 64,891] ∼12.9%, i.e., over 3-times more populated than the 14,472 × 71,917 replicated gene-cell expression matrix overall (4.0% occupancy rate, as previously stated). Put in perspective, the ∼5.2M non-zero UMI tallies within the 623 × 64,891 integrated profiler block represent ∼0.5% of the allowable 14,472 × 71,917 real estate inside the replicated gene-cell expression matrix at the start of the analysis, and 0.07% if considering all 521,628 barcodes expressing replicated genes prior to barcode filtering; similarly, the total ∼7.2M UMIs tallied within the 623 × 64,891 integrated profiler block represent ∼10.4% of the total 69.5M UMIs represented in the replicated gene-cell expression matrix, and less than 7% of the total UMIs aligned in the study.

## Discussion

### Merits of scRNA-Seq Data Analysis as a Latent Variable Extraction Problem

In addition to housekeeping genes that satisfy basic survival needs, different cell populations within a multicellular system also express specialized genes that perform key roles. Historically, cell types are defined around specialization genes whose expression is detected reliably and with the largest variation among individual cells. Following similar logic, the governing principle behind SALSA is also that of parsimony: SALSA explores transcript counts from sc-RNAseq expression matrices, extracts a facultative subset of highly variable genes, and anchors differential single-cell expression analysis around them. Afterward, SALSA advances statistical metrics to qualify measurement reliability across facultative genes, ending with minimal sets of reproducible and cell type-specific Profiler genes to validate independently with bioinformatics-free assays.

SALSA, like other recently reported tools for unguided bioinformatic inferencing of hierarchical associations in biology (Cong et al., 2016; Moussa and Mandoiu, 2018), leverages the concepts behind latent semantic analysis (LSA) methods: the concepts of “genes” and “single cells” found in scRNA-seq data can be thought of as interchangeable with the concepts of “terms” and “documents” in natural language processing algorithms (Luhn, 1958; Salton and Buckley, 1988; Sparck-Jones, 2004; Wu et al., 2008). Both SALSA and LSA perform eigenvalue optimization driven by explicit count data in ultra-sparse matrices using “local” measures of relative frequency for gene/term counts in a cell/document, such as UMI-per-thousand (UPT) or count-per-document total scaling. Additionally, both SALSA and LSA implement “global” weight systems to adjust for the overall frequency of a gene/term vs. all order gene/terms detected anywhere. SALSA and LSA differ in how they compile the preponderance of detected gene/term counts into a useful statistical kernel. In LSA, “global” weights are used to adjust for the incidence of terms throughout a *known* corpus of documents ahead of inferential testing, with the inverse document frequency being the most commonly used “global” weighting statistic. In the SALSA workflow, normalized expression values are estimated from counts of UMIs which must initially be deduplicated, disambiguated, and ascribed to an *unknown* number of single cells. These values are inferred from a pool of observed barcodes and then must be empirically transformed into linearized and normally distributed metrics via generalized linear modeling (Nelder and Wedderburn, 1972; Aitkin and Clayton, 1980; Bullard et al., 2010; Hansen et al., 2011; Oberg et al., 2012; Li and Tibshirani, 2013; Law et al., 2014; Finotello and Di Camillo, 2015; Li and Bushel, 2016; Lozoya et al., 2018).

The implementation of SALSA as a latent variable extraction problem confers two major advantages to scRNA-seq data analysis: it reduces the number of genes needed for inferential testing and increases statistical robustness. By handling the data in this way, we introduce expression transformants that lend single-cell DEG extraction with statistical compliance. In addition, with this approach we can utilize highly efficient and widely available multivariate analyses algorithms that rely on linearity and homoscedasticity assumptions, such as ANOVAs and hierarchical clustering.

### Extending Latent Variable Extraction Methods Like SALSA to scRNA-Seq Analyses

To date, the predominant approach to circumvent scRNA-seq data sparsity is by assembling a single gene × cell expression matrix (regardless of experimental replication) in which empty data blocks, or “dropouts,” are artificially filled in with zeros (Shalek et al., 2013; Wu et al., 2014; Zilionis et al., 2017; Zhang et al., 2018). Instead, SALSA combines highly efficient SVD-driven algorithms for sparse matrix imputation and latent variable extraction techniques per individual biological replicate, which do without artificial zero-inflation, yields smaller gene expression files, and cuts down on the computational footprint required by conventional scRNA-seq data post-processing workflows (Baglama and Reichel, 2005; Picelli, 2017; Haghverdi et al., 2018; Qiu et al., 2018; Zhang et al., 2018). Critically, the methodological enhancements in SALSA prioritize expression data from genes expressed with enough diversity and prevalence—e.g., abundant in some cell subsets within a multicellular specimen but not others—as well as consistently across replicates, that they are more likely to be detected by alternative and less bioinformatics-dependent benchtop techniques in targeted biomarker confirmatory screens.

Experimentally, the probability of capturing transcripts that encode cell type-specific proteins from individual cells within a cell type-specific phenotype is not only stochastic, but also in an active “trade-off” against available intracellular protein stocks (Raj et al., 2006; Liu et al., 2016; Moulana et al., 2018; Hausser et al., 2019; Larsson et al., 2019). In scRNA-seq data, this phenomenon presents as gene × cell expression matrices with ultra-sparse contents and high dropout rates (Mohammadi et al., 2018). Some argue that sparse expression matrices populated only with 1-valued count data suffice to yield statistical insight (Zhang et al., 2018). In our study, we further show that even in reference scRNA-seq benchmark datasets (Macosko et al., 2015; Zheng et al., 2017) which we analyzed and integrated satisfactorily by SALSA, gene × cell expression matrices have near-unary structure, i.e., almost all accrued count data having values of 1, and dropout rates well over 90% of the gene × cell matrix real estate. Given this backdrop, we anticipate SALSA can analyze other scRNA-seq datasets meaningfully if their gene × cell expression matrices show similar information content densities, and may even improve on predictive biomarker extraction from scRNA-seq experiments in the future as transcript retention rates rise with newly enhanced scRNA-seq benchtop chemistries (Di et al., 2020).

SALSA differs from other scRNA-seq workflows in the way it exploits gene representation rates. In other workflows, the inability to dissect expression differences between cell subsets derives from large differences in their UMI totals, which inflates normalized expression rates in single cells with low UMI counts and dilutes them in cells with high ones. We argue that it is critical to consider gene representation rates not only because of the general sparsity of scRNA-seq datsets, but also due to fundamental differences in what defines a DEG in bulk vs. single-cell scales. In bulk RNAseq, the contribution of transcripts from individual cells to a grand total within a cell conglomerate is “averaged out” and compared to those from other cell conglomerates as a continuum. In scRNA-seq data, “average” expression differences between cell conglomerates can result from having all cells from one group expressing less transcripts than all cells in another, having transcripts scattered unevenly or in different proportions between two cell groups that express them, or a combination of both cases. In SALSA, we seek to bridge between bulk and single-cell scales by weighing normalized per-cell expression rates with cluster-level representation rates for each individual gene. By doing so, scRNA-seq data is collapsed into “pseudo-bulked” RNA-seq data compartments, defined by single-cell clusters, and akin to performing independent RNA-seq assays on sorted cell subpopulations. Once again, this approach defines a cell sorting strategy that can be corroborated experimentally using bioinformatics-free methods.

### SALSA: A Bridge Between Exploratory scRNA-Seq Analysis and Biomarker Discovery Assays

The SALSA workflow helped us infer which single-cell transcriptomes in both PBMC 3K and Macosko’s mouse retina DropSeq datasets fell into classes of overarching cell profiles; each of these profiles exhibited a transcriptional signature driven by a core group of DEGs. We show that matrix focusing prioritizes regions within scRNA-seq data harboring the most informative subset of DEGs within the dynamic range of differential expression measurements. Because SALSA defines matrix focusing thresholds parametrically, it allows for systematic replication of statistical analysis between researchers. In all, SALSA minimizes the volume of scRNA-seq data needed for reproducible statistical analysis.

From an experimenter’s perspective, SALSA’s stratification of detected genes highlights important considerations to interpret scRNA-seq data more effectively; it also offers one key warning: without proactive precision benchmarking of sequencing output, the risk of committing experimental resources into validating bioinformatics artifacts grow, e.g., cell types and expression markers based on scRNA-seq data unduly burdened with measurement noise. As the single-cell genomics field keeps moving forward, technologies become more affordable, and datasets get larger, bioinformatic filters that guard against measurement noise during scRNA-seq pattern extraction grow even more relevant. In SALSA, this task is realized in the form of Profiler genes, which represent the top prospective candidates to validate scRNA-seq-based predictions on the bench.

In broad terms, performing matrix focusing helps channel computational resources to the “most variable gene” fraction in the expression matrix, calculate measurement error rates, and establish signal-to-noise thresholds empirically (we don’t know of any existing pipelines that do so). Once filtered against noise, the focused dataset is used in unsupervised clustering and tested for differential expression analysis. From that perspective, the driving purpose for SALSA differs from other imputation-driven scRNA-seq pipelines—such as MAGIC (van Dijk et al., 2018), DeepImpute (Arisdakessian et al., 2019), scImpute (Andrews and Hemberg, 2018), or SAVER (Huang et al., 2018) to mention some—that render a prospective non-sparse expression matrix; instead, SALSA profiles information densities inside scRNA-seq expression matrices (in both per-gene and per-barcode basis) with the available sequenced data to infer the best-candidate subspace that drives unsupervised single-cell clustering based on differential gene expression. SALSA is not conceived as an imputation workflow, but as an information maximization one to coerce scRNA-seq into a cell type-specific marker discovery scheme that exploits imputation-driven clustering (e.g., IRLBA) to prioritize easy-to-catch transcripts and circumvent computational hurdles that arise when dealing with sparse data.

From strictly statistical perspectives, the benefits of tying scRNA-seq analysis to cell type-specific markers with large expression differences have been noted: genes less prone to scoring as “false-positives” by significance testing between cell types using imputed expression matrices are differentially expressed gene candidates with large effect sizes (Andrews and Hemberg, 2018)—in the context of SALSA, those represent the facultative gene subset. By aiming scRNA-seq data interrogation toward facultative genes shared across independently assayed biological specimens, SALSA increases the probability of devoting analytical resources to extracting batch-insensitive sets of “true” replicated and agnostically determined cell type-specific markers. Our results also show it is possible to devise standard best-practices for reproducible scRNA-seq analysis and validation. Therefore, we conclude that the greatest asset of the SALSA workflow is its ability to recognize explicit transcriptional patterns across independent biological replicates, by stratifying detected genes, and from a fraction of the accrued sequencing data that existing scRNA-seq pipelines use.

Based on our observations, the most logical question that arises is also the most intriguing: if it is possible to get meaningful biological insight without “breaking the bank” on sequencing depth, how can we tell between “shallow” and “sufficient” scRNA-seq experiments? The answer is by no means absolute, because it depends on the purpose of the assay—a scRNA-seq experiment in a heterogeneous tissue aimed at finding cell type-specific biomarkers should not require full-exome coverage; a scRNA-seq study to pinpoint differentially expressed genes with alternative splicing in the same biological scenario has no choice. The one certainty is that no single scRNA-seq experiment alone can answer any or all types of biological questions at once and, like other bioinformatics-driven tools, must be corroborated by experimental evidence.

By reframing scRNA-seq analysis as a latent variable scheme with formal reproducibility metrics, we reveal that sparsity-handling data mining strategies with small computational footprints like SALSA can extract testable biological insights through data focusing strategies. Most strikingly, and under the assumption that sparse scRNA-seq data is inevitable, our findings imply that currently recommended sequencing depths for scRNA-seq assays may be excessive—or even wasteful—for experiments meant as hypothesis-generation tools. In time, savings on sequencing expenses per scRNA-seq test could be reinvested to run multiple independent specimens per scRNA-seq study, thereby helping biological replication become the norm for single-cell “omics” at large. Bottom line: SALSA was developed to enhance insight and maximize utility from expensive scRNA-seq data in a world with limiting resources.

## Data Availability Statement

Publicly available datasets were analyzed in this study. This data can be found here: https://s3-us-west-2.amazonaws.com/10x.files/samples/cell/pbmc3k/pbmc3k_filtered_gene_bc_matrices.tar.gz, and NCBI Gene Expression Omnibus (GEO) under accession GSE63473.

## Author Contributions

OL conceptualized and implemented the analytical methodology described in the study, performed data analysis, and conceived data visualization strategies. OL and KM wrote the initial manuscript drafts leading to submission. BP compiled and curated scRNA-seq data. JL-L and HY supervised the selection of scRNA-seq datasets to validate and test the statistical approach described herein. All authors contributed to the design of the study and interpretation of the results, as well as revised, read and approved the submitted version.

## Conflict of Interest

The authors declare that the research was conducted in the absence of any commercial or financial relationships that could be construed as a potential conflict of interest.

## Footnotes

1 https://support.10xgenomics.com/single-cell-gene-expression/datasets

## Supplementary Material

Supplementary Figure 1 Statistical overview and implementation of the SALSA workflow. **(A)** Parametric focusing in the SALSA workflow. Quantile fitting of a P_C_-P_D_ mixture model and a heavy-tailed projection model on per-barcode or per-gene coverages is used to estimate parametric factors and calculate “inlier” coverage bounds. **(B)** Graphical representation of “inlier” coverage bounding.

Supplementary Figure 2 Topographs for 55 landmark and supplemental expression markers of blood cell types, as detected in the PBMC 3K dataset.

Supplementary Figure 3 Profiler genes enriched in cell majors B, F, and G of the PBMC 3K dataset, based on multinomial logistic regression of weighed expression rates.

Supplementary Table 1 List of 166 genes in the PBMC 3K dataset with multi-count UMI alignments (4 or more counts) among gene × cell data-positive expression matrix fields.

Supplementary Table 2 List of 150 overrepresented genes in the Macosko’s mouse retina DropSeq dataset with multi-count UMI alignments (4 or more counts) among gene × cell data-positive expression matrix fields.
