# Supplementary material for: Patterns, Profiles, and Parsimony: Dissecting Transcriptional Signatures From Minimal Single-Cell RNA-Seq Output With SALSA
Source: Front Genet. 2020 Oct 9;11:511286. doi: 10.3389/fgene.2020.511286 (PMC7586319; doi:10.3389/fgene.2020.511286)
Supplement: Supplementary file 1 [file Data_Sheet_1.PDF]

SUPPLEMENTARY MATERIALS

Patterns, Profiles, and Parsimony: dissecting transcriptional signatures from minimal single-cell RNA-seq output with SALSA

Oswaldo A. Lozoya[1,2,*], Kathryn S. McClelland[1,3], Brian N. Papas[4], Jian-Liang Li[4], and Humphrey H-C Yao[3]

[1] *These authors contributed equally to this work*

[2] *Genomic Integrity & Structural Biology Laboratory, National Institute of Environmental Health Sciences, National Institutes of Health, Research Triangle Park, North Carolina, USA*

[3] *Reproductive and Developmental Biology Laboratory, National Institute of Environmental Health Sciences, National Institutes of Health, Research Triangle Park, North Carolina, USA*

[4] *Integrative Bioinformatics Support Group, National Institute of Environmental Health Sciences, National Institutes of Health, Research Triangle Park, North Carolina, USA*

** Author for correspondence:*

Oswaldo A. Lozoya

Tel: +1 984-287-3979

Email: oswaldo.lozoya@nih.gov

Supplementary Discussion

Experimental errors and distributional features for single-cell RNAseq data with different barcoding strategies. A challenge in calculating read counts in all modalities of RNAseq arises when any two reads that map to the same genomic coordinates are detected in the same library. One solution is to append a unique sequence "handle" to reverse transcribed copies of mRNA molecules during cDNA assembly; such a unique molecular identifier (UMI) is usually added during oligonucleotide synthesis within reverse transcription primers by subsequent rounds of single random base pair addition. Introducing UMIs in reverse transcription primers ensures the same UMI is common to all PCR copies from a single cDNA template; later, UMIs help distinguish whether two reads with highly homologous sequences that share the same genome alignment were: a) derived from two distinct cDNA molecules; or b) degenerate PCR copies from the same cDNA template that differ due to base pair integration errors.

In single-cell RNAseq, reads are pooled by their presumed cell of origin (which is designated by barcode sequences) and mapped uniquely to the reference genome. Then, reads are tallied per mapped gene and assigned to individual fields in a gene×cell matrix describing the total read counts mapped to each individual gene (rows) under each captured cell barcode (columns), which we refer to as the "hash matrix" denoted by $\boldsymbol{R}$. For that reason, it is critical that the tally in $\boldsymbol{R}$ is representative of the initial pool of unique mRNA molecules, and that "double-counting" is prevented – which can occur when cDNA templates giving rise to matching PCR copies cannot be traced back to their original mRNA molecule. It follows that when the number of starting mRNA molecules is low, the probability increases that many fragments in the final sequencing library will be PCR copies derived from a single cDNA template. Also, if the sequencing library consists of randomly cleaved UMI-encoding fragments of cDNA copies amassed by PCR, then sequenced reads may be detected that map to slightly different genomic ranges (i.e. PCR copies of each initial cDNA template cleaved into slightly different fragments) while sharing the same UMI (i.e. derived from the same mRNA molecule).

In this context, use of UMIs is particularly relevant to the assembly of single-cell RNAseq libraries, which originate from limiting amounts of mRNA molecules per cell – with only a small fraction of those contributing to cDNA synthesis reactions. The limited yield of cDNA templates derives in low-complexity libraries with a limited mass of distinct fragments. Given mass and size constraints that dsDNA libraries must meet to load successfully onto sequencing flow cells, low-complexity libraries usually undergo successive rounds of purification steps for size selection (e.g. SPRI-based size selection) and removal of oligonucleotide adapter sequences and/or PCR primers. Doing so, however, carries with it an inevitable loss of library mass; the usual remedy against those losses is adding PCR amplification rounds in between purification steps. The hope is to increase the probability of having each initial mRNA template represented in the final sequencing library more than once – by creating multiple copies of fragments derived from the original cDNA templates – to counterbalance mass losses during library purification. For those reasons, the usual outcome after sequencing a low-complexity single-cell RNAseq library is a pool of dsDNA reads composed almost exclusively (>95%) of PCR copies from a small set of UMI-bearing cDNA templates – representing only fragments from cDNA copies that were retained across the entire specimen-to-sequencer hands-on workflow.

As in any other NGS technique, DNA artifacts may contaminate a sample and, on occasion, integrate UMI handles or NGS sequencing adapters needed for sequencing at any step in the specimen-to-sequencer hands-on workflow. In the case of RNAseq, these artifacts may include primer dimers, ambient RNA, or impurities in PCR consumables; specifically, for single-cell RNAseq, mRNA molecules or nucleic acid debris from broken cells that disperse in suspension media are not only templates for cDNA synthesis but also artifacts, because their appended barcodes cannot trace back to one cell of origin. If (when) they occur, any barcoded DNA artifacts become sequencing-ready dsDNA templates in the library that may add to the total counts of detected reads.

In bulk RNAseq assays, where a single barcode annotates transcripts from thousands of cells at once, contributions by DNA artifacts are usually outweighed by the overall diversity of distinct mRNA

molecules present. However, the detriment of such artifacts to single-cell RNAseq data is much more substantial. Unfortunately, most bioinformatic pipelines to populate ***R*** matrices from UMI-deplexed reads do not discriminate between UMIs shared by many sequenced fragments and UMIs detected only once – in other words, UMIs are counted all the same in data post-processing, regardless of whether they were added to the library at the start (e.g. mRNA reverse transcription) or in between steps (e.g. dsDNA contaminants) of the specimen-to-sequencer hands-on workflow. In effect, this also means that UMI-harboring artifacts can impact each field in ***R*** matrices in at least two ways: by distributing heterogeneously across barcodes, or by harboring barcodes of their own. These matters are complicated further by the fact that total UMI counts accrued from individual cell barcodes across detected genes (or vice versa) vary, meaning that an equal number of dsDNA counts added to different cell barcodes does not have the same impact on their expression measurement uncertainty when their total counts are different – i.e. once normalized, the "statistical error" from dsDNA artifacts is different. For all those reasons, a pre-filtering step is needed *even before quantification of single-cell gene expression* to recognize *bona fide* barcodes and UMIs representative of mRNA molecules tractable to their individual cells of origin, while discarding UMI-harboring artifacts that map to the reference genome successfully – some of which do so by mere chance.

In principle, one may anticipate whether the apportionment of dsDNA artifacts in data from a single-cell RNAseq library will lean towards an all-around "smeared UMI noise" mixed with *bona fide* UMIs across multiple cell barcodes, or towards dsDNA artifacts harboring barcodes of their own. Such bias would depend on both the experimental approach to single-cell indexing and the quality of the single cell suspension used for library assembly; presumably, this inference may also work the other way around: the distribution of UMIs per detected barcode in a single-cell RNAseq data set is also a probabilistic fingerprint of the experimental technique and sample conditions used to synthesize a sequenced single-cell RNAseq library. This *a priori* inference is key to devising a frequentist pre-filtering approach to minimize the impact of dsDNA artifact counts to single-cell expression analysis. To do so, recognizing

how barcoding noise and artifacts manifest in overall UMI distributions is key, and depends on which one of (at least) two broadly classified methods is chosen to couple single cells to individual barcodes: by droplet-based encapsulation or combinatorial indexing.

Currently, the most affordable droplet-based encapsulation systems rely on dual split-flow microfluidic devices that feed two separate incoming aqueous suspensions – i.e. cells and barcoding oligonucleotides – into a junction and down a single channel; the coalescing flow is met downstream by a third incoming cross-flow of an immiscible fluid – e.g. oil – that "pinches" the aqueous stream to form single micelles of equal volume. To assign a shared barcode to all transcripts captured from the same cell, oligonucleotides with the same underlying barcode must be packaged into single delivery depots and appended to cDNA copies of RNA templates during reverse transcription. Such barcode depots are built by synthesizing reverse transcription primers onto immobilizing substrates, or carriers, that include randomly integrated nucleotide sequences using successive single-base addition rounds. In this manner, the number of nucleotides in barcodes determines the number of distinct sequences that can be generated; hence, the probability of two or more carrier particles sharing the same random barcode lessens as more nucleotides are stringed onto fewer immobilizing carriers (since the number of possible nucleotide sequences increases) and all but vanishes once the number of random barcodes is greater than the number of supplied carrier particles. Afterwards, random UMI sequences are added to all carriers at once, followed by functional reverse transcription primers – e.g. poly(dT) sequences complementary to poly-A tails found in mRNA transcripts – such that all oligonucleotide molecules immobilized to each single carrier share the same barcode, yet each contains a UMI of its own, and can prime cDNA synthesis from RNA templates. In this manner, each cDNA copy that enters the library assembly workflow is tagged with information about the carrier that captured its source RNA molecule but remains distinguishable from cDNA copies of RNA molecules that hybridized to other oligonucleotides on the same carrier. Today, the most widely used carriers consist of surface-functionalized porous resins or oligo-laden hydrogels and are suspended in buffered solutions containing reverse transcription enzymes and detergents to lyse the

cells that carriers are to be encapsulated with. The purpose is to ensure that once lysed within droplets, single cells release RNA templates to be used for compartmentalized cDNA synthesis reactions on the surface of co-encapsulated barcode carriers, and that the necessary ingredients to carry out reverse transcription are available inside every droplet.

When using a dual split-flow microfluidic device to generate single-cell RNAseq libraries, the probability of initiating uniquely barcoded cDNA synthesis reactions using RNA templates from a single cell is bounded by the probability that a flowing single and unique barcode carrier coincides with the contents of a single cell upon encapsulation – which requires that cells and carriers both reach the junction separately, one of each at a time, and whole. Assuming the arrivals of non-lysed single cells and barcode carriers to an encapsulation junction are both mutually independent Poisson-distributed events, the maximum theoretical probability of singlet collision events (i.e. the probability of a single cell coinciding with a single carrier inside the same droplet) is equal to $1/e^2$, or no more than $\sim$13.5% of all generated droplets containing at least one of the two, and occurs when the average feed rates of cells and carriers match. Conversely, the theoretical minimum proportion of droplets with either no carriers or only carriers, no cells or only cells, multiple carriers that coincide with more than one cell in the same droplet ("multiplets") or vice versa, or empty (i.e. only aqueous media from cell and carrier suspensions) is at least ~86.5% of all droplets produced – i.e. over 6 times the maximum number of expectable singlets. In practical terms, the product of a single-cell encapsulation run is a heterogeneous colloid that consists of mostly multiplets (whose barcodes tag the contents of many cells each) and "empty" droplets (whose contents can only supply trace DNA contaminants, ambient RNA, or cell debris to cDNA synthesis reactions) along with a much smaller fraction of true singlets overall; therefore, most barcodes detected upon sequencing are to be discarded during data processing. Even then, it is relatively simple to recognize barcoded artifacts in a droplet-based single-cell RNAseq library – whether they randomly map to a reference genome (with the lowest UMI total counts overall) or originate as cDNA copies of ambient RNA from lysed cells (with diluted UMI total counts) – as they amount to most of the detected barcodes, and

have the lowest sequencing representation; therefore, barcoded artifacts fall at the lower end of quantile plots for total UMI counts per barcode. Conversely, because their proportion in the library is much smaller but have larger and relatively stable total UMI counts each, "true" single-cell barcodes show up as a "late" plateau in quantile plots. Multiplets are found further up the barcode representation range and exhibit a progression in total UMI counts away from the single-cell plateau in the quantile plot.

Often, supply rates of cells and carriers (or their concentrations) are manipulated to minimize production of multiplets and obtain a final colloid composed of only singlets or empty droplets, such that each sequenced barcode can be assigned to either class during post-processing simply based on having many or few total UMIs, respectively. However, using mismatched supply rates of cells and carriers lowers singlet collision rates dramatically, thereby increasing the proportion of empty droplet barcodes that harbor low UMI totals each (as they tag only DNA artifacts or ambient RNA). This approach can become a double-edged sword during data processing: in the one hand, the distribution of UMI totals per barcode from empty droplets can be leveraged as a minimum of read counts expected per singlet; in the other hand, an overrepresentation of empty droplets in the colloid risks leading to a library composed almost exclusively of empty barcodes, and producing a dataset of only artifacts. This trade-off can only be evaluated empirically, as transcription rates in single cells vary between experimental replicates, cell types, and biological specimens. Also, the ability to distinguish between singlet and empty barcodes based on their total UMI counts depends on how many artifacts like ambient RNA molecules and cell debris are present in the mixing flows. Therefore, the purity of buffers and reagents, handling of biological specimens, and integrity of single-cell suspensions is critical to discriminating between empty barcodes and singlets among an unknown set and number of supplied barcode carriers.

Barcoding in combinatorial indexing (or "split-pooling") starts with *in situ* cDNA synthesis reactions, such that cells or nuclei are used as encapsulation units in the library assembly workflow. In principle, this method relies on reverse transcription primers and enzymes having molecular weights smaller than size exclusion limits of organellar membranes, so they can diffuse in and reach RNA templates;

conversely, RNA templates larger than those exclusion limits cannot exit. Supplying reagents and nucleotides in excess can also impose an osmotic gradient, thereby enhancing this unidirectional exchange. When performed on isolated nuclei or fixed cells, this transport mechanism is passive, thus requiring no microfluidics devices. Once inside, reagents and primers can be activated to integrate index sequences within cDNA molecules inside individual cells; furthermore, if the integrity of cells or organelles is maintained, multiple rounds of indexing can be carried out where indexed cells from one multi-well plate can be retrieved, resuspended, and delivered at random into different multi-well plates for additional indexing rounds. This iterative process results in compounded barcodes, consisting of combinations of known indices appended in successive split-pool rounds, that cDNA templates from a single cell all share and can be decoded during sequenced data post-processing. Therefore, the number of possible index combinations determines a finite number of single-cell compounded barcodes that can turn up in a single-cell sequencing library and depends exclusively on the number of split-pool rounds carried out while using a set number of indices. In practical terms, this also means that the number of split-pool rounds can be chosen in advance to generate more index combinations than the number of cells to be sequenced, all but guaranteeing that the probability of multiple cells sharing the same compounded barcode is negligible.

Above all, the main advantage of combinatorial indexing techniques is that the underlying indices and the number of compounded barcodes they can assemble are fixed and known in advance. This feature of combinatorial indexing also suggests that if the numbers of single cells to be indexed is not only less, but of a similar order of magnitude than the number of barcodes as well, then artifacts are unlikely to harbor barcodes of their own. Instead, in combinatorial indexing, UMIs appended to artifacts will most likely smear across all barcodes as a background signal that contributes to total UMI counts across the board. In that sense, artifacts behave as a detection threshold to distinguish barcodes whose total UMI make-up is predominantly derived from single-cell RNA templates instead of trace contaminants, PCR concatemers, or ambient RNA from lysed cells. Such a threshold can be estimated by parametric fits of

total UMI counts per barcode to a truncated probability function and would be applicable to all barcodes at once. For that reason, any barcodes with sequencing representation over the threshold qualify as single cells, comprise most of the detected barcodes, and appear as an upshot in quantile plots of total UMI counts per barcode (since sequencing representation is usually similar among true single cells vs. artifacts).

Still, the desired number of sequenced cells is a critical parameter to consider when devising an experimental design, whether a droplet-based or a combinatorial indexing approach is chosen. As the number of PCR cycles required to generate sufficient library mass for sequencing necessarily rises with lower cell numbers, PCR fidelity errors accumulate more in specimens containing fewer cells; thus, the base integration errors in the library increase, and degeneracies in barcode and UMI sequence replication start piling up – hindering the purpose of UMI technology altogether. In other words, the probability of misrepresenting UMI sequences is directly linked to the accumulated number of PCR cycles needed to assemble and sequence the library itself. In practice, PCR error "catch-up" in low-complexity single-cell RNAseq libraries means that deeper sequencing in hopes of raising the total UMI counts per barcode may be inherently risky – or even detrimental – to a faithful interpretation of differential gene expression between single cells within a sequenced specimen. To what extent does oversaturation of library complexity in a sequencing run shifts towards production of reads carrying spurious barcodes and UMIs that mismatch those of their original templates (and resemble "new" data) is still a problem for NGS technologies across the board. In lieu of robust corrective measures to interpret "true" UMIs apart from degenerate ones *a posteriori*, it is our view that larger cell numbers, use of ERCC spike-ins, or empirical determination of *quantum sufficit* sequencing depths for different types of specimens all remain the most practical strategies to optimizing bioinformatic analytical pipelines and enhancing reproducible differential expression analysis at the single-cell level.

Stratification of LSTNRs and single-cell clustering in the PBMC 3K data set. After honing the PBMC 3K dataset to a facultative block containing ~1.3M data-positive fields of gene×cell UMI counts,

we then performed differential expression analysis using the SALSA workflow (Fig. 2D). From the facultative block (1.3M data-positive fields, 57.2% of stack) we identified ~76% of genes as SGs (2,519 of the 3,305 genes; Table 1 and Fig. 6A). These genes accounted for the subset of facultative genes that showed statistically significant variation among the 7 prospective clusters, once resolution and within-cluster representation rates were accounted for in combination (Fig. 2D). Going further, SALSA identified an increasingly robust and more statistically stringent subset of LSTNR genes from within SGs, and it did so without barcode or gene dropouts at a 78.4% SG-to-LSTNR data retention rate for the PBMC 3K data set (Table 1). Of note, both the SG and LSTNR blocks represent minute fractions of the original expression matrix available real state, with a 2.3% and 1.8% matrix span respectively.

The precision-benchmarked subset of 2,419 LSTNR genes represents a set of candidate biomarkers to distinguish cells from different lineages by their transcriptional signatures – which are yet to be determined at this stage of the SALSA workflow. We approached this problem by gathering LSTNR-only data, and then we inferred 7 "cell major" groupings via IRLBA re-clustering of GLM-projected expression rates (Fig. 2D). Looking ahead, we realized that when interrogating expression matrices dominated by 1-valued gene×cell count data, how many cells in one cell type express a gene compared to another cell type is perhaps the only differential metric at hand to distinguish them. Thus, we also recorded representation rates of LSTNR genes within cell majors, a metric of how often a LSTNR gene is detected in cells within a major, and equal to the proportion of cells with any number of UMIs aligned to a LSTNR gene per major.

The frosty plot for the PBMC 3K data using SALSA-based gene stratification is shown in Fig. 6A. By sequentially "stripping" statistical comparisons from a 2,419-member list of LSTNR genes from the PBMC 3K data set we sifted the pool down to: a) 1,244 DEGs whose Log_2FC pairwise differences between cell majors are statistically significant and mutually exclusive (DEGs); of which b) 464 showed differences greater than the SNR=1 noise benchmark (DEGREEs); including c) 462 exhibiting statistically distinct and mutually exclusive differences that are insensitive to stochastic incidence of transcripts, or

representation, among cell types (Profilers). Notably, even though the number of retained gene×cell count data fields dropped as the number of genes decreased between strata (Table 1) our stratification approach led to a substantial improvement on information density. Ultimately the 462×2,700 Profiler block represents ~0.5% of the gene-cell matrix allowance, however this span is over 3-times more populated as a subset than the gene-cell expression matrix overall (outlined in Table 1). These results suggest that facultative gene stratification retains underlying transcriptional profiles of single cells, thereby pointing to SALSA successfully extracting a parsimonious subset of testable, agnostically defined candidate biomarkers.

After performing single-cell clustering at the Profiler-stratum level, we first assessed the relative density of data in each of the 7 cell majors, originally labelled A through G. Several intricacies became immediately apparent – for example, not only were the number of cells different among cell majors, but also the distribution of total UMI coverages in each was not even-handed. Based on their clustering dendrogram (Figs. 6B and 6C), all 7 majors that we defined were grossly derived from three core stems: A through D represented one extreme, F and G were situated opposite, and E constituted a lone-standing intermediate stem. In the first stem, only majors A through C, which accounted for slightly over half of all inferred cells, showed similar total UMI coverage distributions among them (roughly 1,000 – 3,000 total UMI per cell; Fig. 6D). Log_2FC expression levels for A through C were in the low-to-mid quantiles among accrued profiler-stratum count data overall (about 0.7 – 1.5 log-fold relative to the bulk-wise average; Fig. 6C). Meanwhile, major D was substantially skewed towards lower total UMI coverages (about 500 – 2,500 total UMI per cell; Fig. 6D), and mid-to-high Log_2FC expression quantiles (up to 2.8 log-fold vs. bulk). Interestingly, based on net Log_2FC values, normalized expression rates in major D were about twice as high as those from the A-to-C ensemble. This is seemingly consistent with the mismatch between both ensembles in total UMI coverage, and to which count data each are normalized against. This observation implies that traditional normalized expression scores at the gene×cell level, such as UPT rates, track with total count coverage rather than intensity of expression. Accordingly, cell major

E in the intermediate branch showed lower expression values relative to the A-C ensemble in the first stem by about a factor of 2. This was consistent with cells in major E carrying roughly twice as many total UMIs the A-C ensemble (about 2,000 – 5,000 total UMI per cell; Fig. 6D). Put simply, these data demonstrate that single-cell expression metrics by normalized-per-coverage rates alone are misleading.

Using IRLBA-based single-cell clustering within the SALSA workflow illustrates how interpretation of expression matrices can go awry if based solely on expression scoring without account for representation or precision benchmarking. For the PBMC 3K dataset, these risks are most evident in the F-G stem: based on hierarchical clustering (Figs. 6B and 6C), majors F and G are more closely related to each other than to any other cell majors, yet their total UMI coverages are at opposite extremes (500 – 2,000 and 2,000 – 16,000 total UMI per cell in F and G, respectively; Fig. 6D). In a seeming contradiction, data in major F aligns to the most Profiler genes while, at the same time, exhibits the lowest per-cell coverage among all majors; major G data make-up for Profiler genes is exactly the opposite.

Peripheral blood cell types in PBMC 3K inferred using SALSA. To examine whether Profiler-based unsupervised clustering tracked with transcriptional signatures from peripheral blood subpopulations, we focused on expression data from a reference subset of 15 "landmark" genes encoding 14 widely recognized protein markers (Figs. 6C, 6E and 6F; Table 2). To do so, we inspected within-cluster distributions of Log_2FC expression levels relative to the whole-specimen collective. To visualise the clustering data in 2D, we also devised "topographs" consisting of neighbour-joining trees overlayed with a non-parametric quantile heatmap of expression scores weighed by representation rates. We use these topographs to simultaneously highlight differences in the intensity and predominance of expressed genes among clusters of like cells (Figs. 6F and S2).

Single-cell clustering based on Profiler genes in the 3K PBMC dataset revealed 7 distinctive single-cell clusters (Figs. 6B and 6C) split into two overarching transcriptome categories; the first category containing the A-D ensemble and the intermediate E stem (2,071 cells combined), and the second containing the F-G stem (629 cells combined). Within the first transcriptome category, majors A, C, D

and E all expressed CD124(IL4R), a marker shared by both T and B lymphocytes (Tan et al., 2011; Zola et al., 1993) (Fig. S2). While majors A, C and E had an expression profile characteristic of T cells, major D was distinguished by rich CD19+/CD20(MS4A1)+ expression consistent with a B cell phenotype (Fig. 6E) (Chauhan, 2016; Gustafson et al., 2015; Henson et al., 2012; Hu et al., 2018; Mahnke et al., 2013; Sallusto et al., 1999; Zhao and Davies, 2010). Cell major B was one of the richest CD4+ subpopulations in the entire dataset (Fig. 6C). Based on the overall pattern of landmark gene expression and other supplemental markers, such as Fc-γ receptors (Fig. S2), we determined that the transcriptional profile of cells in major B was consistent with granulocytes (Gustafson et al., 2015; Lin and Lore, 2017; Pyzik et al., 2015). Together, lymphoid-derived T cells (majors A, C and E) and B cells (major D) accounted for 1,864 cells; this contribution is consistent with the reported 4:1 ratio, for cells of lymphoid vs. myeloid origin in the source PBMC stock (AllCells®, 2019)

To further split out the functions of majors A, C and E we exploited the unique characteristics of lymphoid-derived T cells subclasses. Transcriptionally, lymphoid-derived T cells exhibit differences based on their maturation state, regulatory vs. inflammatory roles, and degrees of antigen-presenting and antigen-discovery competence (Chauhan, 2016; Churlaud et al., 2015; Du et al., 2006; Henson et al., 2012; Khattri et al., 2003; Liu et al., 2006; Mahnke et al., 2013; Sallusto et al., 1999; Zhao and Davies, 2010; Zola et al., 1993). However, transcriptional differences between majors A, C and E in the PBMC 3K data set (depicted using violin plots; Fig. 6E) were too subtle to be matched to discrete T cell subtypes based on normalized expression rates alone. In this context, inspection of gene representation rates proved critical in assigning lineage identity to cell majors; here, topograph visualisation outperforms violin plots by simultaneously revealing log-fold expression, representation rates, and the location of expressing individual cells in clustering maps for a gene of interest (Fig. 6F). We confirmed, our identification of major D as the B cell subpopulation agreed with the near exclusive expression of additional B cell population markers (Fig. S2) (Hu et al., 2018; Karnell et al., 2014). Next, based on a panel of landmark genes (Table 2) we identified majors E, A and C as naïve, memory and cytotoxic T cells respectively; this

is in agreement with varying degrees of enrichment for additional T cell maturation markers (Fig. S2) (Ahlers and Belyakov, 2010; Churlaud et al., 2015; Hu et al., 2018; Khattri et al., 2003; Yagi et al., 2004).

Arrangement of cell subpopulations within the first transcriptome category, encompassing majors A, C, D and E, revealed that SALSA-based unsupervised clustering can successfully reconstruct matches between cell types and their coordinated biological functions. For example, we found that majors C and D had the closest transcriptomic similarity among all clustered cell subpopulations (Fig. 6B). Notably majors C and D correspond to cytotoxic T cell and B cell lineages which are mature subpopulations with closely linked and converging physiological roles (Karnell et al., 2014; Mahnke et al., 2013; Sallusto et al., 1999; Tan et al., 2011; Zola et al., 1993). Likewise, the inferred proximity between memory T cells in major A, and granulocytes in major B in the clustering map (Fig. 6B) suggests a significant interaction between those two cell types. Physiologically this relationship is widely recognized; subsets of granulocytes are known to perform antigen-presenting roles that help coordinate CD4+ T cell activation (Gustafson et al., 2015; Lin and Lore, 2017; Pyzik et al., 2015).

The second transcriptome category constituted majors F and G. This was puzzling because apart from strikingly different total UMI coverages, we also found that expression of blood cell-type markers in major G cells had the strongest similarities with granulocytes (major B) in the first transcriptome category (Bednar et al., 2014; Hambleton et al., 1996; Shi et al., 2004; Wakabayashi et al., 2006) (Figs. S2 and S3). However, major G cells clustered apart from granulocytes in major B and were also distinct in critical ways; for example, major G cells showed much higher expression levels of monocytic and macrophage-enriched genes CD16(FCGR3A) and MS4A4A, respectively (Fig. 6B, 6C and 6E) (Hu et al., 2018; Mandl et al., 2014; Sanyal et al., 2017). We concluded that cell major G represents a combined pool of monocyte-derived subtypes including monocytes, macrophages, and mono-derived dendritic cells. This conclusion was based on two key factors: an implied myeloid origin, like that of antigen-presenting granulocytes in major B, and transcriptional enrichment for genes involved in pathogen recognition processes that are characteristic of leukocytes from the innate immune system (Du et al., 2006; Gustafson

et al., 2015; Hambleton et al., 1996; Hu et al., 2018; Kowal et al., 2004; Mandl et al., 2014; Sanyal et al., 2017; Shi et al., 2004).

Given this backdrop, we surmised that if clustering proximity in the second transcriptome category evokes converging physiologies then majors F and G should both present key ontologies. To test this, we asked whether majors F and G shared a minimal set of abundantly detected Profiler genes which: a) were absent from all other cell majors; and b) significantly enriched for critical innate immunity processes or molecular functions. After surveying weighed expression rates by multinomial logistic regression, we found that both majors F and G prominently expressed the Profiler genes HIST1H2AC, PF4, PPBP, and SDPR(CAVIN2). These genes all participate in anti-parasitic NETosis processes, mediate leukocyte chemoattraction and degranulation, and are regulated by calcium-sensitive protein kinase C activity (Baig et al., 2009; Brinkmann and Zychlinsky, 2012; Cullen, 2003; Diaz-Godinez and Carrero, 2019; Lian et al., 2009; Liverani et al., 2018; Walz et al., 1989). Importantly, most other cells in the PBMC 3K dataset did not express any of these Profiler genes or exhibit their associated ontologies (Fig. S3).

Still, the transcriptomic profiles of cells in major F were evidently distinct from monocytic cell types. Therefore, we used key defining features to derive the identity of the cells in major F: 1) their strong ontological relationship with monocytic cell types, 2) their relative frequency in the data set of ~9% of the total (247 single cells), and 3) their inferred regulatory functions linked to innate immunity within PBMC fractions. From these features we deduced that cells in major F correspond to lymphoid-derived natural killer (NK) cells (AllCells®, 2019; Gustafsson et al., 2008; Hanna et al., 2004; Martinet et al., 2012; Pokkali et al., 2009; Poli et al., 2009; Romee et al., 2013).

Finally, the segregation of T cell subtypes, B cells, and antigen-presenting granulocytes under the same transcriptome category when using the SALSA workflow was consistent with an underlying and powerful biological feature: those four cell types constitute the adaptive immune system. Conversely, the second transcriptome category depicted the main players in the innate immune response: NK cells and monocyte-derived cells. We found that unsupervised clustering of single cells inferred by SALSA

recapitulated the expression patterns of traditional marker genes and proportions of cell types expected from PBMC specimens without data preconditioning. Thus, by performing single-cell profiling on the "silver" standard PBMC 3K dataset, we demonstrate the core strengths of the SALSA workflow. With SALSA, a minimal fraction of well-resolved expression data from agnostic Profiler genes successfully sorted like cells, recapitulated experimentally demonstrated transcriptional signatures, and retained latent linkages that evoke converging physiologies among interconnected cell types.

Supplementary Methods

Unifying probabilistic mixture model for single-cell sequencing depth parameterization. Understanding how artifact representation differs between droplet- v. combinatorial-based techniques has important implications to inferring whether UMI-containing reads represent cells or artifacts. As discussed previously, the expectable noise fingerprints in UMI representation of artifacts are almost opposite between droplet-based and combinatorial-based techniques from a statistical perspective: whereas artifacts tend to accrue their own barcodes in droplet-based libraries, they contribute background reads to all barcodes indiscriminately in combinatorial indexing libraries instead. Hence, artifact representation differences between techniques should *in principle* be clearly discernible by displaying quantile plots of total UMI counts per barcode.

In practice, though, capturing such stark contrasts between separate libraries assembled with either droplet- or combinatorial-based techniques is experimentally possible only if the biological specimen to be sequenced is truly dissociated into single cells, which do not clump together over time, while ensuring that ambient RNA from premature cell lysis in suspension medium is minimal. This is rarely the case, and often results in quantile plots of total UMI counts per barcode that resemble a mix of both the droplet-based and split-pool-based methods, with a bias towards the actual technique used. In that sense, total UMI distributions in single-cell RNAseq libraries can be thought of as mixtures of two extreme cases: the theoretical droplet-based and combinatorial-based profiles. Therefore, a 2-distribution parametric mixture

model is the simplest statistical model to fit empirical distributions of total UMIs from single-cell RNAseq libraries experimentally produced using either technique.

Consider a single-cell RNAseq library $\boldsymbol{R}$ described by a $B \times G$ tensor of sequenced UMIs, each encoding one of B unique barcodes and uniquely mapped to one of G total genes represented in a sequenced library. Thus, $\boldsymbol{R}$ is the total set of unique sequencing read counts in the library, or "hash matrix", such that

$$\boldsymbol{R} = R_{ij}$$

describes (in Einstein notation) a tally of nonduplicate templates detected in the library under the ith captured barcode and mapped to the jth gene in the reference genome. Thus, given an all-ones column vector $\mathcal{X}_k = 1$ of size $G \times 1$,

$$b_i = R_{ik}\mathcal{X}_k$$

represents the total counted and uniquely identified sequences in the library carrying the ith barcode – i.e. the total UMI counts in the ith barcode.

Now, let us define a probabilistic mixture model of b_i with $N \geq n$ parametric probability functions, such that

$$w_n \delta_{nk} = 1$$

and

$$\mathrm{P}(b_i \leq x) = w_n \mathrm{P}_n \delta_{nk} + \varepsilon_n \delta_{nk}$$

given the set of positive weights w_n for individual parametric probability functions P_n, where δ_{nk} is the Kronecker delta, and ε_n are regression error terms. Therefore, a 2-distribution parametric mixture model of theoretical droplet-based combinatorial-based probability functions is given by

$$\mathrm{P}(b_i \leq x) = w_\mathrm{D} \cdot \mathrm{P}_\mathrm{D} + w_\mathrm{C} \cdot \mathrm{P}_\mathrm{C} + \varepsilon_D + \varepsilon_C$$

where

$$w_\mathrm{D} = 1 - w_\mathrm{C}$$

and P_D and P_C denote probability functions for theoretical (i.e. without multiplets) droplet-based and combinatorial-based libraries, respectively.

If we assume that the theoretical droplet-based library consists mostly of barcodes with low UMI counts assigned to artifacts that transitions afterwards into a small proportion of barcodes with higher UMI counts for singlets, then its theoretical distribution P_D for total UMIs per barcode b_i can be represented by a Weibull probability function bounded at $x > 0$ such that

$$P_D(b_i \leq x) \sim \mathrm{Wb}(x; \beta, \alpha) = \frac{\beta}{\alpha} \cdot \left(\frac{x}{\alpha}\right)^{\beta-1} \cdot \exp\left[-\left(\frac{x}{\alpha}\right)^{\beta}\right]$$

where P_D refers to a theoretical droplet-based library, and $\beta > 0$ and $\alpha > 0$ are Weibull shape and scale parameters, respectively. Furthermore, $\beta \geq 1$ if the inference for the ith barcode to represent a singlet improves as b_i rises.

The closely related Fréchet probability function bounded at $x > 0$ is suitable to represent the distribution of b_i in the theoretical combinatorial index-based library, in which barcodes are assigned to single cells and accrue total UMI counts well over artifacts randomly spread throughout; it is defined as

$$P_C(b_i \leq x) \sim \mathrm{Fr}(x; \mu, \nu) = \frac{\mu}{\nu} \cdot \left(\frac{x}{\nu}\right)^{-\mu-1} \cdot \exp\left[-\left(\frac{x}{\nu}\right)^{-\mu}\right]$$

in which P_C refers to the probability function of a theoretical combinatorial index-based library, and $\mu > 0$ and $\nu > 0$ are Fréchet shape and scale parameters, respectively. Also, $\mu \geq 1$ if the probability P_C for any i^{th} barcode to approach the minimum value of $b_i > 0$ is asymptotically small – i.e. P_C speeds upwards at $b_i \gg 0$.

Hence, assuming negligible error $\lim_{n\to\infty}(\varepsilon_n \delta_{nk}) = 0$, it can be shown the P_C-P_D parametric mixture model for b_i in a single-cell RNAseq library can be algebraically reduced to

$$P(b_i \leq x) = [P_D] \cdot [1 - w_C \cdot (1 + P_{C*D})]$$

where

$$\mathrm{P}_{\mathrm{C*D}} = \frac{\Omega}{\mathrm{A}} \cdot \mathrm{X}^{\Omega-1} \cdot \exp\left[-\frac{1}{\alpha^{\beta}} \cdot \left(\frac{\mathrm{X}^{\Omega} - \mathrm{A} \cdot \mathrm{X}}{\mathrm{A}}\right)\right]$$

is a parametric probability distribution of the exponential family bounded at $\{\mathrm{X} = x^{\beta}\} > 0$ and expressed in terms of composite parameters $\Omega = -(\mu/\beta)$ and $\mathrm{A} = 1/(\nu^{\mu} \cdot \alpha^{\beta})$. Put simply, the $\mathrm{P_C}$-$\mathrm{P_D}$ mixture model predicts that, for any single-cell RNAseq library, the distribution of b_i rises suddenly from a low-count detection limit defined by artifacts (consistent with a Fréchet distribution), exhibits a transition phase, tails off as b_i values keep rising (consistent with a Weibull distribution), and stops as the expected values of b_i reach an improbably high maximum value corresponding to multiplets.

The purpose of fitting a $\mathrm{P_C}$-$\mathrm{P_D}$ mixture model is to tease out which detected barcodes correspond to single cells based on their total UMI counts b_i. As mentioned earlier, we assume that single cells reside somewhere past the transition phase in the cumulative distribution of b_i and into the tail-off range towards the maximum value of b_i. In that sense, one could infer – based on b_i values alone – that barcodes representing single cells reside in an interphase $b_{\mathrm{Low}} \leq b_i \leq b_{\mathrm{High}}$ coalescing a Fréchet-dominated low-count domain (b_i surges when going from artifacts to single cells) and a Weibull-dominated high-count domain (b_i surges again when going from single to multiple cells). Thus, the expectation is that a $\mathrm{P_C}$-$\mathrm{P_D}$ mixture model will exist such that the underlying $\mathrm{P_C}$ and $\mathrm{P_D}$ distributions dominate at opposite extremes of the b_i domain. If so, the scale parameters α and ν for $\mathrm{P_D}$ and $\mathrm{P_C}$, which denote the inflection points in each surge of the b_i domain, can be used as fiducial marks to help infer limits in barcode coverages characteristic of single cells – i.e. $\nu \sim b_{\mathrm{Low}}$ and $\alpha \sim b_{\mathrm{High}}$. Accordingly, the shape parameters β and μ estimate how steep those surges are when moving away from the ν and α fiducial marks, respectively.

To minimize the likelihood of admitting artifact barcodes when defining the single-cell interphase $b_{\mathrm{Low}} \leq b_i \leq b_{\mathrm{High}}$, we trim the b_i domain inwards from the low-end fiducial mark ν to define b_{Low}. We reason that, being a Fréchet inflection point between noise and single-cell data, barcodes with $b_i = \nu$ are statistically indistinguishable from either. Thus, to prioritize certainty in single cell assignment, we

impose a systematic shift such that $b_{\text{Low}} > \nu$. In doing so, we take three features into account: the relative Fréchet behavior contribution w_{C}, a combined steepness metric $\mu \cdot \beta$, and the proportional enrichment α/ν between fiducial marks for the P_{C}-P_{D} mixture model such that

$$b_{\text{Low}} = \nu \cdot \left(\frac{\alpha}{\nu}\right)^{\frac{w_{\text{C}}}{\mu \cdot \beta}}$$

corresponding, in logarithmic scale, to a weighed baseline shift from the low-end fiducial mark ν adjusted for steepness and not to encroach the high-end fiducial mark α:

$$\ln(b_{\text{Low}}) = \ln(\nu) + w_{\text{C}} \cdot \left(\frac{1}{\mu \cdot \beta}\right) [\ln(\alpha) - \ln(\nu)].$$

We recognize a similar case can be made at the high end of the b_i spectrum – with some important modifications. In our interpretation of the b_i probabilistic model, the transition going from artifacts to single cells reminisces a Fréchet-dominated probabilistic behavior. Thus, by fitting a single Frechét parametric distribution P_{Fr} to the entire b_i domain, while taking into account the contribution of low-count artefacts, such that

$$\text{P}_0(b_i \leq x) \sim \text{Fr}(x; \mu_0, \nu_0) = \frac{\mu_0}{\nu_0} \cdot \left(\frac{x}{\nu_0}\right)^{-\mu_0 - 1} \cdot \exp\left[-\left(\frac{x}{\nu_0}\right)^{-\mu_0}\right]$$

one can project a parametrically determined b_{High} single-cell upper bound for P_0 corresponding to a linear shift over the scale factor ν_0 (a measure of the artefact-to-noise turning point), systematically weighed by the shape constant $\mu_0 > 1$, and mimicking a multiplet-free experiment:

$$b_{\text{High}} = \nu_0 + 2 \cdot \mu_0 \cdot (\overline{x_0} - \nu_0)$$

where $\overline{x_0} = \nu_0 \cdot \Gamma(1 - 1/\mu_0)$ is the expected arithmetic mean of P_{Fr}. Hence, our parametric definition of b_{High} represents a doubling of the effective average gain in b_i values among single cells vs. the baseline signal from artifacts, or $(\overline{x_{\text{Fr}}} - \nu_0)$, which we adjust by a factor of μ_0 – i.e. the compactness of the histogram of observed b_i values – to correct for projected dispersion in the P_{Fr} distribution.

An important property of this definition for b_{High} is that it is not only anchored – since fitting P_{Fr} depends on the amount of artifact barcodes – but also self-ballasted: higher values of μ_0 bring the tails of predicted P_{Fr} histograms closer, shrinking the effective distance (or gain) between the ν_0 baseline and the projected mean $\overline{x_{\text{Fr}}}$, while simultaneously stretching the linear shift between b_{High} and ν_0 by a factor of μ_0. For example, even though values of the projected b_{High} can vary widely in proportion to the baseline ν_0 (between 3-6 times larger for typical $1.5 < \mu_0 < 3$ fitted values) the actual rate of admitted barcodes, or $\mathrm{P}_{\text{Fr}}\left(b_i/\nu_0 \leq b_{\text{High}}/\nu_0\right)$, remains largely the same across the board (e.g. between 93rd-97th percentile for typical $1.5 < \mu_0 < 3$ fitted values). In other words, our parametric definition of the pass-fail criterion b_{High} to distinguish singlets vs. multiplets is a frequentist projection that depends on two non-dimensional constants: μ_0 and b_{High}/ν_0. Combined, these properties predict two systematic advantages: a) the fraction of inferred singlets $b_i < b_{\text{High}}$ remains the same for a single-cell library even if re-sequenced to increase coverage, as artifact and singlet values both scale with ν_0; and b) multiplets with $b_i \geq b_{\text{High}}$ are a systematic outlier subset, accounting for roughly the same fraction of detected barcodes overall, even for independent single-cell libraries that do not share the same fitted estimates for μ_0 or ν_0.

References

Ahlers, J.D., and Belyakov, I.M. (2010). Memories that last forever: strategies for optimizing vaccine T-cell memory. Blood *115*, 1678-1689.

AllCells® (2019).

Baig, A., Bao, X., Wolf, M., *et al.* (2009). The platelet protein kinase C substrate pleckstrin binds directly to SDPR protein. Platelets *20*, 446-457.

Bednar, F., Song, C., Bardi, G., *et al.* (2014). Cross-desensitization of CCR1, but not CCR2, following activation of the formyl peptide receptor FPR1. J Immunol *192*, 5305-5313.

Brinkmann, V., and Zychlinsky, A. (2012). Neutrophil extracellular traps: is immunity the second function of chromatin? J Cell Biol *198*, 773-783.

Chauhan, A.K. (2016). Human CD4(+) T-Cells: A Role for Low-Affinity Fc Receptors. Front Immunol *7*, 215.

Churlaud, G., Pitoiset, F., Jebbawi, F., *et al.* (2015). Human and Mouse CD8(+)CD25(+)FOXP3(+) Regulatory T Cells at Steady State and during Interleukin-2 Therapy. Front Immunol *6*, 171.

Cullen, P.J. (2003). Calcium signalling: the ups and downs of protein kinase C. Curr Biol *13*, R699-701.

Diaz-Godinez, C., and Carrero, J.C. (2019). The state of art of neutrophil extracellular traps in protozoan and helminthic infections. Biosci Rep *39*.

Du, X., Tang, Y., Xu, H., *et al.* (2006). Genomic profiles for human peripheral blood T cells, B cells, natural killer cells, monocytes, and polymorphonuclear cells: comparisons to ischemic stroke, migraine, and Tourette syndrome. Genomics *87*, 693-703.

Gustafson, M.P., Lin, Y., Maas, M.L., *et al.* (2015). A method for identification and analysis of non-overlapping myeloid immunophenotypes in humans. PloS one *10*, e0121546.

Gustafsson, K., Ingelsten, M., Bergqvist, L., *et al.* (2008). Recruitment and activation of natural killer cells in vitro by a human dendritic cell vaccine. Cancer research *68*, 5965-5971.

Hambleton, J., Weinstein, S.L., Lem, L*., et al.* (1996). Activation of c-Jun N-terminal kinase in bacterial lipopolysaccharide-stimulated macrophages. Proc Natl Acad Sci U S A *93*, 2774-2778.

Hanna, J., Bechtel, P., Zhai, Y.F*., et al.* (2004). Novel insights on human NK cells' immunological modalities revealed by gene expression profiling. J Immunol *173*, 6547-6563.

Henson, S.M., Riddell, N.E., and Akbar, A.N. (2012). Properties of end-stage human T cells defined by CD45RA re-expression. Curr Opin Immunol *24*, 476-481.

Hu, Z., Jujjavarapu, C., Hughey, J.J*., et al.* (2018). MetaCyto: A Tool for Automated Meta-analysis of Mass and Flow Cytometry Data. Cell reports *24*, 1377-1388.

Karnell, J.L., Dimasi, N., Karnell, F.G., 3rd*, et al.* (2014). CD19 and CD32b differentially regulate human B cell responsiveness. J Immunol *192*, 1480-1490.

Khattri, R., Cox, T., Yasayko, S.A*., et al.* (2003). An essential role for Scurfin in CD4+CD25+ T regulatory cells. Nat Immunol *4*, 337-342.

Kowal, K., Osada, J., Zukowski, S*., et al.* (2004). Expression of interleukin 4 receptors in bronchial asthma patients who underwent specific immunotherapy. Ann Allergy Asthma Immunol *93*, 68-75.

Lian, L., Wang, Y., Flick, M*., et al.* (2009). Loss of pleckstrin defines a novel pathway for PKC-mediated exocytosis. Blood *113*, 3577-3584.

Lin, A., and Lore, K. (2017). Granulocytes: New Members of the Antigen-Presenting Cell Family. Front Immunol *8*, 1781.

Liu, W., Putnam, A.L., Xu-Yu, Z*., et al.* (2006). CD127 expression inversely correlates with FoxP3 and suppressive function of human CD4+ T reg cells. J Exp Med *203*, 1701-1711.

Liverani, E., Mondrinos, M.J., Sun, S*., et al.* (2018). Role of Protein Kinase C-delta in regulating platelet activation and platelet-leukocyte interaction during sepsis. PloS one *13*, e0195379.

Mahnke, Y.D., Brodie, T.M., Sallusto, F*., et al.* (2013). The who's who of T-cell differentiation: human memory T-cell subsets. Eur J Immunol *43*, 2797-2809.

Mandl, M., Schmitz, S., Weber, C., *et al.* (2014). Characterization of the CD14++CD16+ monocyte population in human bone marrow. PloS one *9*, e112140.

Martinet, J., Dufeu-Duchesne, T., Bruder Costa, J., *et al.* (2012). Altered functions of plasmacytoid dendritic cells and reduced cytolytic activity of natural killer cells in patients with chronic HBV infection. Gastroenterology *143*, 1586-1596 e1588.

Pokkali, S., Das, S.D., and Selvaraj, A. (2009). Differential upregulation of chemokine receptors on CD56 NK cells and their transmigration to the site of infection in tuberculous pleurisy. FEMS Immunol Med Microbiol *55*, 352-360.

Poli, A., Michel, T., Theresine, M., *et al.* (2009). CD56bright natural killer (NK) cells: an important NK cell subset. Immunology *126*, 458-465.

Pyzik, M., Rath, T., Lencer, W.I., *et al.* (2015). FcRn: The Architect Behind the Immune and Nonimmune Functions of IgG and Albumin. J Immunol *194*, 4595-4603.

Romee, R., Foley, B., Lenvik, T., *et al.* (2013). NK cell CD16 surface expression and function is regulated by a disintegrin and metalloprotease-17 (ADAM17). Blood *121*, 3599-3608.

Sallusto, F., Lenig, D., Forster, R., *et al.* (1999). Two subsets of memory T lymphocytes with distinct homing potentials and effector functions. Nature *401*, 708-712.

Sanyal, R., Polyak, M.J., Zuccolo, J., *et al.* (2017). MS4A4A: a novel cell surface marker for M2 macrophages and plasma cells. Immunol Cell Biol *95*, 611-619.

Shi, G.X., Harrison, K., Han, S.B., *et al.* (2004). Toll-like receptor signaling alters the expression of regulator of G protein signaling proteins in dendritic cells: implications for G protein-coupled receptor signaling. J Immunol *172*, 5175-5184.

Tan, C., Taylor, A.A., Coburn, M.Z., *et al.* (2011). Ten-color flow cytometry reveals distinct patterns of expression of CD124 and CD126 by developing thymocytes. BMC Immunol *12*, 36.

Wakabayashi, Y., Kobayashi, M., Akashi-Takamura, S., *et al.* (2006). A protein associated with toll-like receptor 4 (PRAT4A) regulates cell surface expression of TLR4. J Immunol *177*, 1772-1779.

Walz, A., Dewald, B., von Tscharner, V., *et al.* (1989). Effects of the neutrophil-activating peptide NAP-2, platelet basic protein, connective tissue-activating peptide III and platelet factor 4 on human neutrophils. J Exp Med *170*, 1745-1750.

Yagi, H., Nomura, T., Nakamura, K., *et al.* (2004). Crucial role of FOXP3 in the development and function of human CD25+CD4+ regulatory T cells. Int Immunol *16*, 1643-1656.

Zhao, C., and Davies, J.D. (2010). A peripheral CD4+ T cell precursor for naive, memory, and regulatory T cells. J Exp Med *207*, 2883-2894.

Zola, H., Flego, L., and Weedon, H. (1993). Expression of IL-4 receptor on human T and B lymphocytes. Cell Immunol *150*, 149-158.
