# Supplementary figures and images for: Patterns, Profiles, and Parsimony: Dissecting Transcriptional Signatures From Minimal Single-Cell RNA-Seq Output With SALSA
Source: Front Genet. 2020 Oct 9;11:511286. doi: 10.3389/fgene.2020.511286 (PMC7586319; doi:10.3389/fgene.2020.511286)

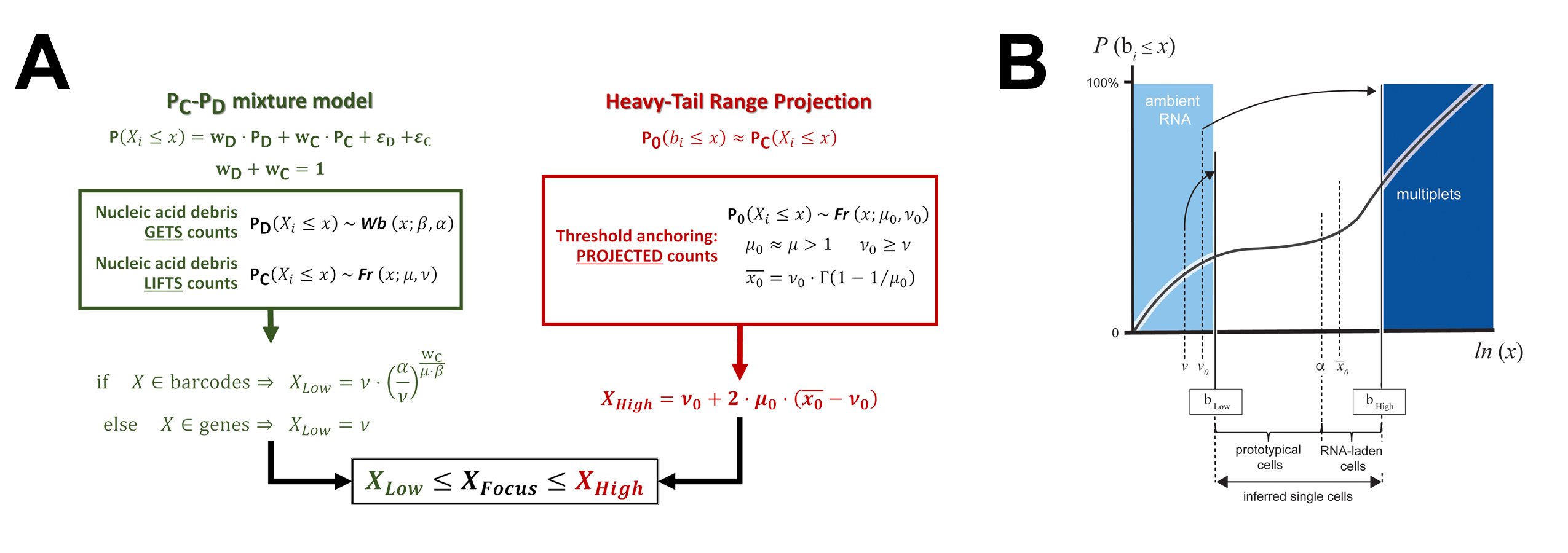

Supplement: Supplementary Figure 1 — Statistical overview and implementation of the SALSA workflow. (A) Parametric focusing in the SALSA workflow. Quantile fitting of a PC-PD mixture model and a heavy-tailed projection model on per-barcode or per-gene coverages is used to estimate parametric factors and calculate “inlier” coverage bounds. (B) Graphical representation of “inlier” coverage bounding. [file Image_1.JPEG]

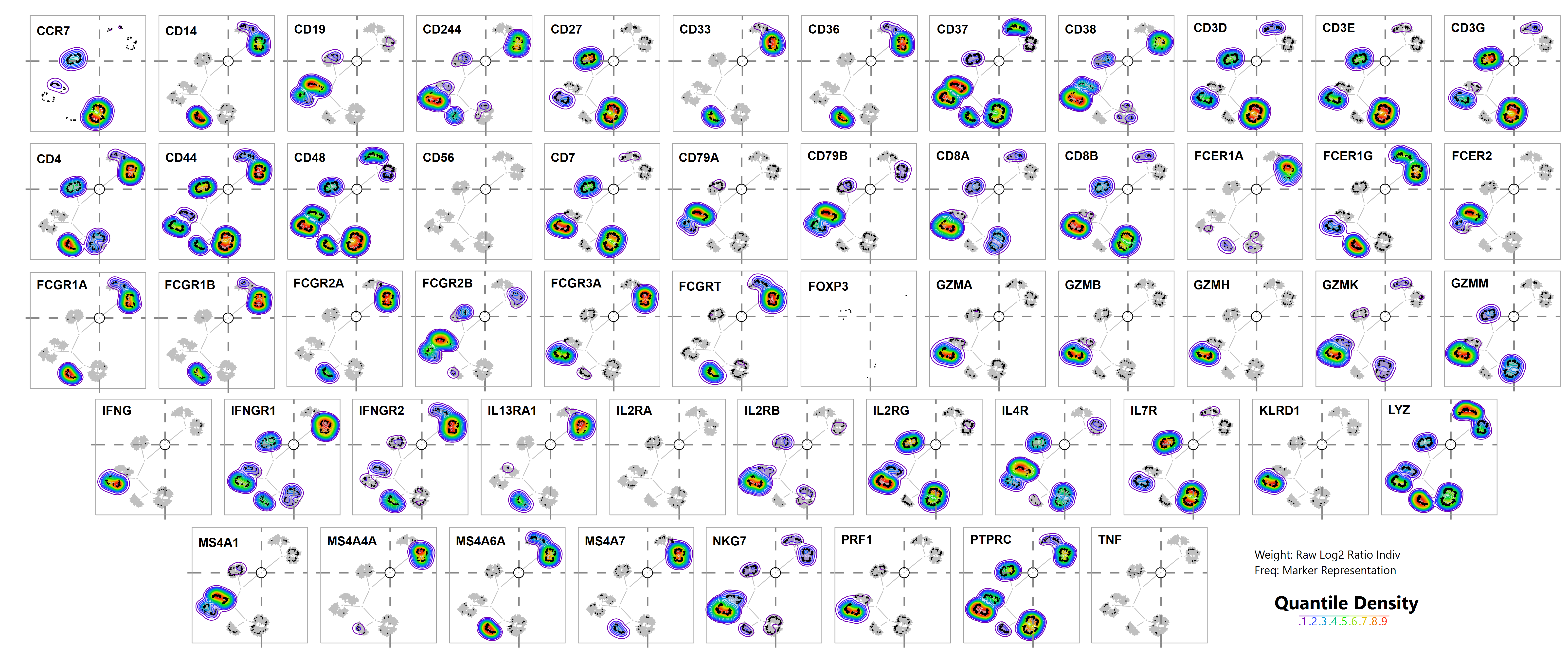

Supplement: Supplementary Figure 2 — Topographs for 55 landmark and supplemental expression markers of blood cell types, as detected in the PBMC 3K dataset. [file Image_2.PNG]

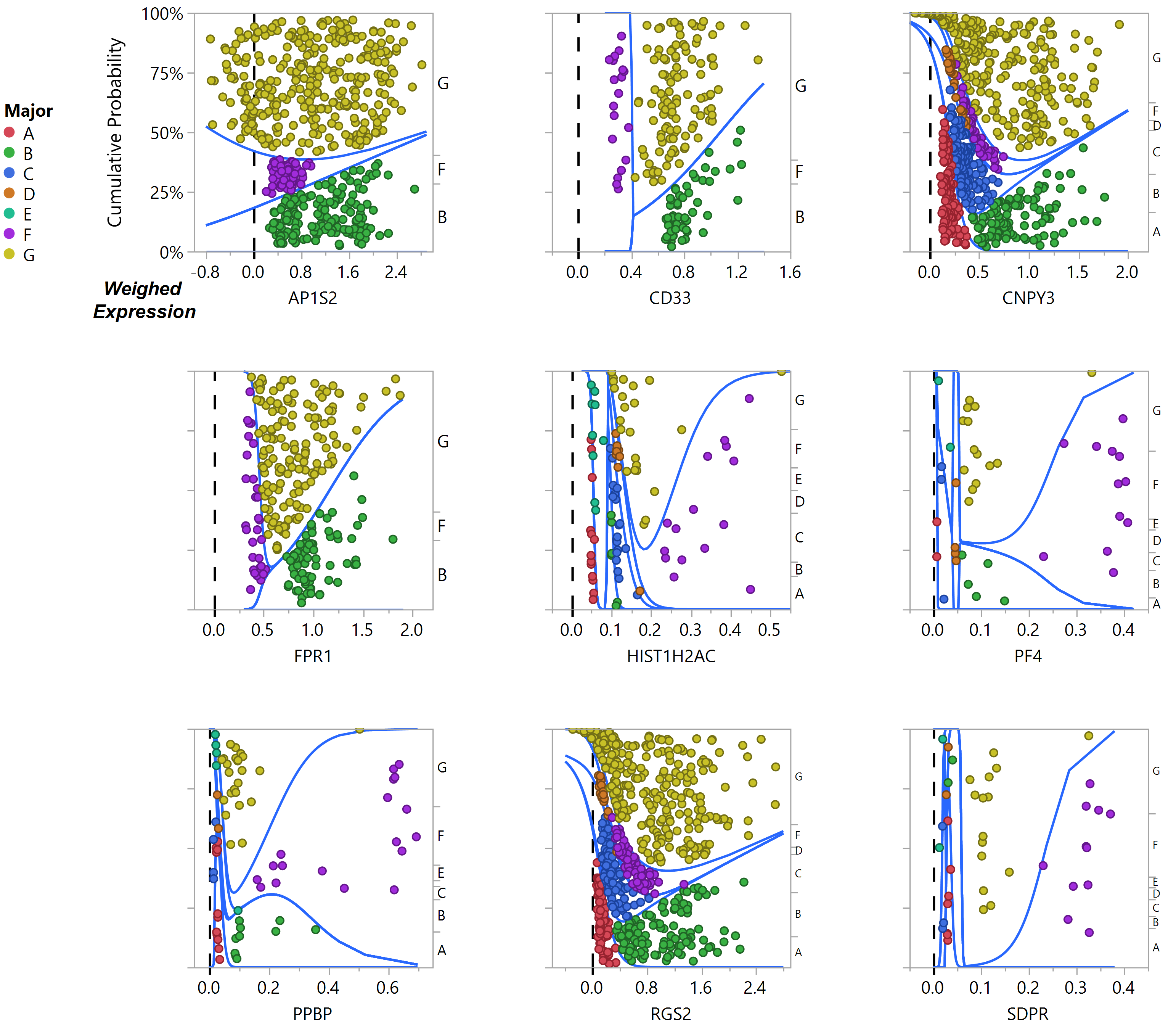

Supplement: Supplementary Figure 3 — Profiler genes enriched in cell majors B, F, and G of the PBMC 3K dataset, based on multinomial logistic regression of weighed expression rates. [file Image_3.PNG]
